# Phosphoproteomic profiling reveals post-translational dysregulation in Huntington’s disease patient-derived neurons

**DOI:** 10.1186/s11658-026-00948-2

**Published:** 2026-06-02

**Authors:** Lea Danics, Chandramouli Muralidharan, Ágnes Varga, Melinda Rezeli, Jeovanis Gil, Anna A. Abbas, Ádám Pap, Andrew S. Park, Marcell Cserhalmi, Emilie M. Legault, Ármin Sőth, Dorina Jamniczky, Roland Zsoldos, Roger A. Barker, Gergely Róna, Janelle Drouin-Ouellet, György Markó-Varga, Zsuzsanna Darula, Karolina Pircs

**Affiliations:** 1 Institute of Clinical Pathophysiology, Semmelweis University, Budapest, Hungary; 2 Hungarian Centre of Excellence for Molecular Medicine—Semmelweis University (HCEMM-SU), Neurobiology and Neurodegenerative Diseases Research Group, Budapest, Hungary; 3 HUN-REN-SU Cerebrovascular and Neurocognitive Diseases Research Group, Budapest, Hungary; 4 Laboratory of Molecular Neurogenetics, Department of Experimental Medical Science, Wallenberg Neuroscience Center and Lund Stem Cell Center, Lund University, Lund, Sweden; 5 HUN-REN-SZTAKI-SU Rejuvenation Research Group, HUN-REN Office for Supported Research Groups (TKI), Budapest, Hungary; 6 Division for Biomedical Engineering, Department of Biomedical Engineering, Lund University, Lund, Sweden; 7 BioMS−Swedish National Infrastructure for Biological Mass Spectrometry, Lund University, Lund, Sweden; 8 Clinical Chemistry, Department of Translational Medicine, Lund University, Lund, Sweden; 9 Single Cell Omics Advanced Core Facility, Hungarian Centre of Excellence for Molecular Medicine, Szeged, Hungary; 10 Laboratory of Proteomics, Complex Molecular and Cell Biology Service Centre, HUN-REN Biological Research Centre, Szeged, Hungary; 11 Faculty of Pharmacy, University of Montreal, Montreal, QC Canada; 12 Centre de Recherche Sur Le Cerveau Et L’apprentissage (CIRCA), University of Montreal, Montreal, QC Canada; 13 MTA-HUN-REN RCNS Lendület “Momentum” DNA Repair Research Group, Institute of Molecular Life Sciences, HUN-REN Research Centre for Natural Sciences, Budapest, Hungary; 14 Cambridge Stem Cell Institute and John Van Geest Centre for Brain Repair, Department of Clinical Neurosciences, University of Cambridge, Forvie Site, Cambridge, UK; 15 Department of Biochemistry and Molecular Pharmacology, NYU Grossman School of Medicine, New York, NY USA

**Keywords:** Huntington’s disease, Phosphorylation, Induced neurons, Post-translational modification, MXRA8, Autophagy

## Abstract

Huntington’s disease (HD) is a fatal neurodegenerative disorder caused by a CAG repeat expansion in the *Huntingtin* gene. Although transcriptomic and proteomic changes have been characterized in patient-derived neurons, the contribution of post-translational modifications, such as phosphorylation, remains poorly understood. Here, we present the first phosphoproteomic analysis by mass spectrometry (P-MS) of human induced neurons (iNs) directly reprogrammed from HD patient fibroblasts. We identified 177 phosphopeptides with significantly altered abundance in HD-iNs, mapping to phosphoproteins associated with key signaling pathways known to be affected in HD, such as splicing and autophagy. By integrating P-MS data with previously published proteomic and transcriptomic data from the same donors, we identified distinct subsets of ON–OFF phosphopeptides that exhibited a complete loss of phosphorylation in either HD- or control-iNs, without corresponding changes at the RNA or protein level. An exception was MXRA8, previously described in glial cells as a mediator of blood–brain barrier integrity and astrocyte-mediated neuroinflammation. This protein showed increased protein abundance despite the absence of phosphorylation in HD-iNs, suggesting a compensatory mechanism. In addition, MXRA8 showed altered protein–protein interactions with lysosomal and metabolic regulators in HD-iNs, highlighting its potential role in autophagy impairment as well as in neurovascular dysfunction. These findings uncover a distinct layer of post-translational dysregulation in HD, suggesting that phospho-switch proteins such as MXRA8 may be candidate effectors of pathology, and thus, site-specific phosphorylation loss may contribute to impaired signaling and proteostasis in human HD neurons.

**Supplementary Information:**

The online version contains supplementary material available at 10.1186/s11658-026-00948-2.

## Introduction

Huntington’s disease (HD) is an autosomal dominant genetic disorder characterized by age-dependent progressive neurodegeneration predominantly in the striatum and in the cortex of the brain [1, 2]. Although HD is considered a rare disorder, studies show a consistent increase in HD incidence and prevalence over the past decades, posing a growing burden on families, healthcare systems, and society [3–6]. HD manifests with progressing cognitive impairment, involuntary movements, psychiatric, metabolic and sleeping problems, and eventually death of the patient typically within about 20 years after the symptoms first appear [1, 7]. Despite decades of research, no curative treatment is currently available.

The disease is caused by expanded cytosine–adenine–guanine (CAG) repeats in one of the alleles of the *Huntingtin* (*HTT*) gene, translated to a mutant HTT (mHTT) protein containing an abnormally long poly-glutamine (polyQ) tract near the N-terminus [2]. This mHTT protein exhibits toxic gain-of-function properties and has a greater tendency to misfold, fragment, and form aggregates when compared with the wild-type protein [1, 2]. However, beyond the toxicity of mHTT in HD, numerous pathways, associated with autophagy, intracellular trafficking, energy metabolism, synaptic function, and protein homeostasis, are disrupted, leading to the pathophysiology and progression of the disease [1, 8]. A better understanding of the molecular changes involved in such pathways in HD could lead to alternate therapeutic interventions that target the affected pathways.

Protein phosphorylation, the most prevalent post-translational modification (PTM), is a critical player in cell signaling pathways and is known to be dysregulated in various neurodegenerative disorders. In addition, dysregulation of phosphorylation has previously been shown to play a pivotal role in the pathology of HD [9–16]. However, there is a lack of in-depth characterization of phosphorylation events in HD, which could offer deeper insights into the altered signaling pathways and help discover potential new therapeutic targets.

Studying HD remains challenging, as it is a human-specific disorder and is strongly influenced by aging, features that are difficult to replicate in conventional cellular or animal models [17–20]. In this study, we performed high-throughput phosphoproteomic analysis by mass spectrometry (P-MS) using a previously established induced neuronal (iN) model derived from HD patient fibroblasts with age- and sex-matched controls, which retains the age-related epigenetic characteristics of the donor [21, 22]. The neuronally enriched iN populations investigated here (hereafter referred to as iNs) represent heterogeneous pan-neuronal cultures and have been extensively characterized in prior studies [21–25]. When integrated with proteomic data from the same cohort in our previous study [18], we observed striking alterations in the phosphoproteome of HD-iNs compared with their proteome profiles, suggesting that PTMs may play a significant role in HD pathology. We identified 46 phosphosites across 43 proteins that were phosphorylated exclusively in either control iNs or HD-iNs, referred to as ON–OFF and OFF–ON phosphoproteins, indicating disrupted phosphorylation regulation of key proteins and pathways.

Strikingly, only one ON–OFF protein in HD-iNs, the matrix remodeling associated protein 8 (MXRA8), showed a complete lack of phosphorylation at a specific site, coupled with a significant increase in abundance at the protein level, indicating a possible compensatory effect to MXRA8 dysfunction. Using a custom-made phospho-specific antibody, we validated this P-MS and MS phenotype of MXRA8 in HD-iNs. Further, we identified several candidate kinases involved in regulating MXRA8 phosphorylation and confirmed the dysregulation of some of these by western blotting (WB). Consistent with these findings, immunohistochemical (IHC) analysis of post mortem HD cortical tissue showed MXRA8 expression in MAP2-positive neurons. Lastly, we found altered MXRA8 binding partners through co-immunoprecipitation (co-IP) coupled with MS, which revealed a novel role in neuronal autophagy regulation and neurovascular function [26].

This study therefore presents the first open-access phosphoproteomic landscape of human HD-iNs. By combining our previously published proteomic analysis with this new phosphoproteomic data using the same HD samples, we have provided a multiomic approach to better understand HD pathophysiology [22]. Detection of ON–OFF proteins such as MXRA8 highlights new mechanistic processes in HD pathology, potentially leading to the identification of novel therapeutic targets and underscoring the translational potential of PTMs.

## Methods

### Fibroblast lines

In this study we used dermal fibroblast samples from a previously published cohort of seven patients with HD and seven age-matched, non-genetically related healthy individuals, as controls (Ctrl) (Table 1) [22]. Fibroblasts were obtained via skin biopsy, as described previously [21]. The skin biopsy samples were obtained from the Huntington’s Disease Clinic at the John van Geest Centre for Brain Repair (Cambridge, UK) and the Fondazione IRCCS, Instituto Neurologico Carlo Besta (Milan, Italy) and used under local ethical approvals (REC 09/H0311/88, IV-2625-1/2021/EKU, IV-1029-1/2022/EKU). The CAG repeat length was defined by Sanger sequencing for both alleles (Laragen Sanger Sequencing Services). The age of cohort ranged between 27 and 66 years old. The CAG repeat length of the *mHTT* allele in the HD patients was between 39 and 45 repeats [27]. All healthy control subjects had CAG repeat lengths below 24 (Table 1).

**Table 1:** Demographic summary of Ctrl and HD patients

ID	Age	Sex	CAG repeats (wild-type/mutant)	Age at onset of HD, years
HD1	28	M	15/39	Premanifest
HD2	31	M	20/45	33
HD3	43	M	17/42	38
HD4	43	M	19/44	36
HD5	47	M	NA/40	Premanifest
HD6	53	M	19/42	Premanifest
HD7	59	M	16/39	33
C1	27	M	17/17	–
C2	30	M	19/24	–
C3	52	F	19/23	–
C4	54	F	15/20	–
C5	61	M	17/23	–
C6	61	F	17/17	–
C7	66	M	24/24	–

### Cell culture

Fibroblasts were kept in Glutamax supplemented high-glucose Dulbecco’s modified Eagle medium (DMEM) (Gibco) containing 10% fetal bovine serum (FBS) (Euroclone or Hyclone) and 1% penicillin–streptomycin (Gibco). Cells were passaged at 80–90% confluency following the protocol described previously [22].

### Lentiviral production and neuronal conversion

Third-generation lentiviral vectors were produced following a previously described protocol [28]. Virus titration was performed by reverse transcription-quantitative polymerase chain reaction (RT-qPCR) and the titer determined as previously described [28]. Virus titers ranged between 2.70 × 10^8^ and 1.86 × 10^9^.

For the direct conversion of dermal fibroblasts into induced neurons, the previously published LV.U6.shREST1.U6.shREST2.hPGK.BRN2.hPGK.ASCL1.WPRE all-in-one transfer vector was used [21]. The construct, available from the plasmid repository, contains two short hairpin RNAs (shRNA) targeting repressing element silencing transcription factor (REST) 1 and 2 after a U6 promoter and transcription factors ASCL1 (Achaete-Scute family bHLH transcription factor 1) and BRN2 (POU class 3 homeobox 2, also known as POU3F2) after the nonregulated ubiquitous phosphoglycerate kinase (PGK) promoter and a Woodchuck hepatitis virus (WHP) post-transcriptional regulatory element (WPRE).

Fibroblasts were plated at a density of ~26,000 cells/cm^2^ on Nunc Delta surface-treated 24-well plates, (Thermo Scientific) pre-coated with poly-L-ornithine (Sigma), fibronectin (Thermo Fisher), and laminin 111 (Biolamina) or tissue-culture-treated T25 and T75 flasks (Thermo Scientific) pre-coated with 0.1% gelatine (Sigma) [22, 25]. Then, 24 h later, the fibroblasts were transduced with the all-in-one lentiviral vector at multiplicity of infection (MOI) 10 or 20 and incubated for 72 h. Neural conversion was continued with growth factors and small molecules containing NDiff medium (Takara Bio) as previously described [21, 22]. iNs were harvested or fixed on day 28 for further experiments.

The neuronal identity, maturation, and purity of the iN cultures were thoroughly characterized by immunocytochemistry for canonical neuronal markers (TAU, MAP2, TUJ1/βIII-tubulin, NEUN, SYN1), neuronal morphology, transcriptomic profiling, global proteomics, and electrophysiological properties consistent with neuronal function [21–25].

### Phosphoproteomic analysis by mass spectrometry

iNs converted in T75 flasks (600,000 fibroblasts plated for conversion per sample) were dissociated as previously described and prepared for phosphoproteomic analysis as follows [22]. The cells were carefully washed off and collected in a tube with Accutase and spun at 400 *g* for 5 min. The supernatant was discarded, and the pellets were washed three times with Dulbecco’s phosphate-buffered saline (DPBS). After the final wash, the supernatant was aspirated, and the pellets were frozen on dry ice and stored at −80 °C until use.

The cell pellets were resuspended in 200 µl lysis buffer [50 mM dithiothreitol (DTT), 2% sodium dodecyl sulfate (SDS), 100 mM tris(hydroxymethyl)aminomethane (TRIS) pH = 8.6, supplemented with Halt Protease and Phosphatase Inhibitor Cocktail (Thermo Scientific)], rested for 1 min on ice and sonicated (20 cycles: 15 s on/off; Bioruptor plus model UCD-300, Diagenode). Reduction and alkylation of disulfide bridges was performed by incubating the samples at 95 °C for 5 min, followed by the addition of iodoacetamide to a final concentration of 100 mM with incubation for 20 min at room temperature in the dark.

Samples were processed using S-Trap Mini Spin Columns (ProtiFi, USA) according to the manufacturer’s instructions. Briefly, samples were acidified by adding phosphoric acid to a final concentration of 1.2%, seven volumes of binding buffer [90% methanol (MeOH) and 100 mM triethylammonium bicarbonate (TEAB), pH = 7.1] was added to the samples, which were then transferred to the S-Traps, and spun at 4000 *g* for 30 s. The trapped proteins were washed three times with the binding buffer. Protein digestion was performed by adding trypsin (Promega Biotech AB) 1:50 (enzyme:protein ratio) in 125 µl of 50 mM TEAB and incubating for 16 h at 37 °C. Peptides were eluted with 0.2% of aqueous formic acid (FA) and 0.2% of formic acid in 50:50 water:acetonitrile. Following speed vacuum concentration, peptides were dissolved in 0.1% trifluoroacetic acid (TFA) and quantified with the Pierce Quantitative Colorimetric Peptide Assay (Thermo Fisher Scientific). In total, 50 μg of peptides were subjected to metal ion affinity chromatography (IMAC) enrichment using ferrum(III)-nitrilotriacetic acid [Fe(III)-NTA] cartridges on the Agilent AssayMAP Bravo platform (Agilent Technologies), as previously described [29]. The enriched phosphopeptides were resuspended in 20 μl of 2% acetonitrile (ACN) with 0.1% TFA after speed vacuum concentration, and 15 μl was injected for each sample on the nanoscale liquid chromatography coupled tandem mass spectrometry (nanoLC-MS/MS) system.

NanoLC-MS/MS analysis was performed on a Dionex Ultimate 3000 RSLCnano Ultra-Performance Liquid Chromatography (UPLC) system coupled to a Q-Exactive HF-X mass spectrometer (Thermo Fisher Scientific). Phosphopeptides were first trapped on an Acclaim PepMap100 C18 trap column (3 µm, 100 Å, 75 µm i.d. × 2 cm, nanoViper) and separated on an EASY-spray RSLC C18 analytical column (2 µm, 100 Å, 75 µm i.d. × 25 cm) using a nonlinear gradient. Solvent A consisted of 0.1% FA, while solvent B contained 80% ACN with 0.08% FA. The flow rate was set to 0.3 μl/min, and the column temperature was 45 °C. The gradient was initiated at 4% solvent B, ramped to 27% over 120 min, increased to 45% over the next 15 min, then rapidly increased to 98% within 1 min, and held at 98% for an additional 5 min. A top 15 data-dependent analysis (DDA) method was applied, where first-stage mass spectrometry (MS1) full scans were acquired with a resolution of 120,000 (at 200 *m/z*), a target automatic gain control (AGC) value of 3 ×10^6^, and a maximum injection time (IT) of 50 ms, using a mass range of 375–1500 *m/z*. The 15 most intense peaks were fragmented with a normalized collision energy (NCE) of 25. Second-stage mass spectrometry (MS2) scans were acquired with a resolution of 60,000, a target AGC value of 1 ×10^5^, and a maximum IT of 120 ms. The ion selection threshold was set to 7.2 ×10^3^ and the dynamic exclusion to 30 s.

Protein identification and label-free quantification was performed using Proteome Discoverer v2.2 (Thermo Fisher Scientific) with SEQUEST HT as the search engine and a human protein database downloaded from UniProt on 2019-01-15. Trypsin was specified as the protease, allowing for up to two missed cleavages. The mass tolerance was set to 10 ppm for MS1 and 0.02 Da for MS2. Carbamidomethylation of cysteine was set as a static modification, while methionine oxidation, phosphorylation on serine, threonine and tyrosine, and protein N-terminal acetylation were included as dynamic modifications. Phosphorylation site scoring was performed using the Post-Translational Modification Site Reconstruction and Scoring (ptmRS) algorithm, applying a site probability threshold of >75%. Peptides and corresponding proteins were identified at a 1% false discovery rate (FDR).

All phosphopeptide intensities were log_2_-transformed and median-centered per sample to correct for differences in sample loading. Technical replicates were averaged, and only peptides quantified in at least four of seven samples in at least one group were retained, yielding 4363 phosphopeptides corresponding to 1493 different proteins. In addition, phosphopeptide intensities were normalized to the corresponding protein abundances using matched global proteome data [22]. Specifically, each phosphopeptide was mapped to its parent protein in the global dataset, and normalization was performed by subtracting the log_2_-transformed protein intensity from the log_2_-transformed phosphopeptide intensity (log_2_[phosphopeptide] − log_2_[protein]). The resulting values represent phosphorylation changes independent of protein expression. Prior to this correction, missing protein intensities in the global proteome were imputed from the lower tail of the intensity distribution to avoid artificially inflating the phospho-to-protein differences. Principal component analyses were performed in Perseus v1.6.5.0.

### Phosphoproteomic gene-centric analysis

Using a combination of Shapiro–Wilk and an unpaired *t*-test on R v4.3.1 [30], a differential abundance analysis was performed on the phosphopeptides, comparing HD-iNs with Ctrl-iNs. All phosphopeptides with a Shapiro–Wilk *p* > 0.05, or with a mean abundance of 0 in either Ctrl-iNs or HD-iNs were considered for the unpaired *t*-test. Subsequently, those with a *t*-test *p* < 0.05 were considered significant. Further, pathway enrichment analysis was performed on the phosphoproteins corresponding to the significantly differentially abundant phosphopeptides, using STRING-db v12.0 [31] on the corresponding website, with a background combining all the proteins detected in the phosphoproteomic data and those in the proteomic data from Pircs et al. [22].

### Phosphosite enrichment analysis

Phosphosite annotated peptide sequences across the differentially abundant phosphopeptides, along with their log_2_-fold change were uploaded onto the PSEA webtool, Kinase Library, on the PhosphoSitePlus website [32–34]. The results were downloaded as tab-delimited files and further analyzed using R v4.3.1. All kinases with a *p*-value <0.05, and those previously identified in the proteomic data from Pircs et al. [22], were considered significant and inspected further.

### Kinase prediction

The phosphosites associated with the ON–OFF phosphopeptides were each manually searched for on the PhosphoSitePlus website, and using the Kinase Library’s kinase prediction webtool, kinases were predicted [32–34]. The kinase prediction results for each phosphosite were downloaded as tab-delimited files and further analyzed using R v4.3.1. All kinases with a site percentile score greater than 90 and previously identified in the proteomic data from Pircs et al. [22] were considered significant and inspected further.

### Immunocytochemistry

Immunocytochemistry (ICC) on iNs was performed using a previously described protocol [21, 22]. Briefly, on day 28, iNs were fixed with 4% paraformaldehyde (PFA) (pH = 7.4) at room temperature for 10 min. After washing with DPBS, iNs were permeabilized with 0.1% TritonX in DPBS for 10 min and then blocked for 30 min with a blocking solution of 5% donkey serum in DPBS. After blocking, primary antibodies diluted in blocking solution were added to the cells and incubated overnight at 4 °C (Table 2). After removing the primary antibodies, the cells were washed twice with DPBS and then incubated for 2 h at room temperature in the dark with fluorophore-conjugated secondary antibodies diluted in blocking solution (Table 2). Secondary antibodies were removed by washing the cells with DPBS, then 4′,6-diamidino-2-phenylindole (DAPI) staining was applied and incubated for 15 min at room temperature in the dark. DAPI staining was removed, and cells were washed with DPBS before high-content automated microscopy analysis.

**Table 2:** List of antibodies used for ICC and IHC

Antibody	Dilution	Species	Supplier	Cat. no.	RRID
TAU (clone HT7)	1:500	Mouse	Thermo Scientific	MN1000	AB_2314654
MXRA8	1:100	Rabbit	Abcam	Ab185444	N/A
MAP2	1:1,000	Chicken	Abcam	AB5392	AB_2138153
MAP2	1:1,000	Guinea pig	Synaptic Systems	188004	AB_2138181
Alexa Fluor^®^ 488 AffiniPure Donkey IgG	1:200	Mouse	Jackson	715-545-150	AB_2340846
Alexa Fluor^®^ 647 AffiniPure Donkey IgG	1:200	Rabbit	Jackson	711-605-152	AB_2492288

### High-content automated screening microscopy

A CellInsight CX5 high-content automated screening (HCS) platform was used for microscopic analysis of the immunostained iNs. By this method, a fast and unbiased analysis can be performed. For the identification of DAPI^+^ and TAU^+^ cells, we used the target activation (TA) protocol of the CX5 HCS software. Images were acquired from 25 or 100 fields on 96-well plates using a 10× or 20× objective, respectively, and wells were included for further analysis in which there were at least 50 valid neurons. The TA protocol defined DAPI^+^ cells by intensity, shape, and area values. Border objects and DAPI^+^ cells which were aggregated, and where single nuclei could not be segmented by the software, were excluded. Within the DAPI^+^ cells, TAU^+^ cells were then defined on the basis of average cell body fluorescent intensity and area. The purity of the iN culture was determined as the fraction of TAU^+^ cells of the total DAPI^+^ cells, and conversion efficiency as the number of TAU^+^ cells over the starting number of fibroblasts used for that conversion [22].

For analyzing MXRA8 staining, the neural profiling protocol was applied with spot detection using a 20× objective on 144 or 529 fields per well of a 96-well plate or 24-well plate, respectively, and wells were included for further analysis in which there were at least 50 valid neurons. First, DAPI^+^ nuclei and TAU^+^ cells were identified as described above, and TAU^+^ cell bodies and neurites were defined as the region of interest (ROI). Then, the average number and area of different dots per cell in TAU^+^ cell bodies and neurites were determined. In addition, a separate spot detection analysis was performed in which the average number and size of MXRA8 dots were quantified specifically within the nuclei of TAU^+^ cells. Border objects and wells with less than 50% valid fields were excluded from analysis.

The fluorescent intensity, shape, and area threshold settings were defined on randomly chosen fields of view from both groups of iNs. The accuracy of the settings was verified by a short pre-analysis on ten images from each well, which was validated by an independent researcher.

### Immunofluorescence on paraffin-embedded human brain sections

The Cambridge Brain Bank provided anonymous, 10-μm thick paraffin-embedded nonconsecutive tissue sections from patients with HD (*n* = 3) and age-matched controls (*n* = 3) known not to have any neurological or psychiatric disorders (Table 3). Cortical tissue was available for all cases. Demographic data was obtained from the Brain Bank. The pathological severity of HD was scored according to the Vonsattel grading system [35]. Brains sections were used under full local ethical approval (CERC-2025-7229; University of Montreal).

**Table 3:** Human post-mortem brain tissue samples

Brainbank ID	Age at death, years	Sex	Pathological grade
H682	40	M	4
H720	68	M	4
H725	58	M	4
C568	69	M	NA
PT149	56	M	NA
PT155	39	F	NA

NA, not applicable; M, male; F, female

Deparaffinized and rehydrated tissue sections were incubated overnight at 4 °C with the following primary antibodies: rabbit anti-MXRA8, guinea pig anti-microtubule-associated protein 2 (MAP2), and chicken anti-MAP2. On the second day, after washing three times with DPBS, cyanine-conjugated secondary antibodies (1:200; Jackson ImmunoResearch Laboratories) were added and counterstained with DAPI (1:1000; Sigma-Aldrich). To reduce tissue autofluorescence, sections were treated with an autofluorescence eliminator reagent (Sigma). Controls included staining after omitting the primary antibodies to distinguish tissue autofluorescence from specific antibody staining. Fluorescent images of 2048 px by 2048 px were acquired with a Nikon AX-R confocal microscope system with a Nikon Spatial Array Confocal (NSPARC) detector equipped with a 20× lens (PLAN APO 20× DIC M/N2). Analyses on the human post-mortem brains were done with NIS Elements Advanced Research (v6.10.02). A signal intensity threshold approach was used for neuronal segmentation on the basis of MAP2^+^ staining. MXRA8^+^ spot count, and signal intensity were quantified using a threshold-based approach and the Segment.ai tool. Briefly, 18 representative images were selected, and MXRA8^+^ spots were manually traced to generate training annotations. Segment.ai was then run for 5000 iterations.

### Custom phospho-antibody design

For phospho-MXRA8 protein identification we ordered a custom rabbit polyclonal phospho-peptide antibody from Biomatik (Ontario, Canada). The antibody recognizes the protein sequence including the phospho-S377 (pS377) phosphosite of MXRA8 protein. The sequence of the designed phosphopeptide is Cys-GGYEYSDQK(pS)GKSKGKD representing the 367–384 residues of human MXRA8, containing an additional Cys at the N-terminus of the peptide for chemical conjugation purposes (pS represents the phosphorylated Ser). A non-phosphopeptide with the same sequence, with the exception of a non-phospho-S377 instead of pS377, was used as negative control. For antibody production a standard 70-day immunization protocol was used. From two immunized rabbits 1 ml immune serum was collected in total, and the isolated antibodies were purified by affinity chromatography. The quality of the antibody was verified by enzyme-linked immunosorbent assay (ELISA) measurement [serial dilution > 1:32,000; optical density (OD) = 1].

### Western blot

For western blot experiments, 200,000 fibroblasts were converted into iNs in a 0.1% gelatine-coated T25 flask for each sample. iNs were harvested on day 28 by disassociating with Accutase (Corning) and then collected with Hank’s Balanced Salt Solution (HBSS) (Gibco) and spun at 400*g* for 5 min. The cell pellets were lysed with radio-immunoprecipitation assay buffer (RIPA) (Sigma) containing a 4% complete protease inhibitor cocktail (PIC) (Roche) and 4% PhosSTOP phosphatase inhibitor (Roche). Cell lysates were collected and incubated on ice for 30 min followed by centrifugation at 10,000 *g* for 10 min at 4 °C to sediment cellular debris. The supernatant was collected and stored at −80 °C until further use. Gel electrophoresis and semi-dry blotting was performed as described previously [36]. The serine/threonine kinase 10 (STK10) WB was made following the manufacturer’s instructions: the primary antibody was incubated for 1.5 h at RT, after washing, the secondary antibody was incubated for 1 h at 37 °C. Primary and secondary antibodies were diluted in a 5% milk blocking solution (Table 4). For blot detection the Immobilon Western Chemiluminescent HRP Substrate (Millipore) was used; blots were incubated for 5 min at room temperature in the dark followed by immediate visualization by Imager2Imager CHEMI Premium Gel Documentation System (VWR). Band intensity was quantified using Fiji software and corrected to the amount of beta-actin reference protein per lane.

**Table 4:** List of antibodies used for WB

Antibody	Dilution	Species	Supplier	Cat. no.	RRID
MXRA8	1:1,000	Rabbit	Abcam	Ab185444	N/A
MXRA8	1:1,000	Rabbit	Biomatik	N/A (custom made)	N/A
MXRA8-pS377	1:1,000	Rabbit	Biomatik	N/A (custom made)	N/A
MAPK9	1:1,000	Rabbit	Cell Signaling Technology	9258T	N/A
STK10	1:500	Rabbit	Proteintech	25471-1-AP	AB_2880096
EEF2K	1:1,000	Rabbit	Invitrogen	PA5-22175	AB_11153234
HRP-conjugated antibody	1:5,000	Rabbit	Cytiva	NA9340	AB_772191
β-actin	1:50,000	Mouse	Sigma-Aldrich	A3854	AB_262011

### Co-immunoprecipitation (co-IP), mass spectrometry, and data analysis

Immunoprecipitation experiments were performed as described previously [37]. Briefly, fibroblasts or iNs were collected into pre-chilled 1.5-ml tubes using Accutase for detachment. The cells were spun down at 300 *g* for 5 min at 4 °C, then the pellet was washed with DPBS and spun down again. The supernatant was removed, and the pellets were snap frozen and stored at −80 °C until further processing.

Next, the cells were lysed in lysis buffer [25 mM TRIS.HCl pH 8.0, 150 mM NaCl, 0.2% Nonidet P-40 (NP-40), 10% glycerol, 1 mM ethylenediaminetetraacetic acid (EDTA), 1 mM ethylene glycol tetraacetic acid (EGTA), 2 mM MgCl_2_, 0.5 mM 1,4-dithiothreitol (DTT) and 5 mM *N*-ethylmaleimide (Sigma-Aldrich) containing 5 U/ml TurboNuclease (Accelagen)] for 30 min on ice aided with a 1 min burst of sonication (Joanlab UC30D). The insoluble fraction was removed by centrifugation (20,000 *g* for 10 min at 4 °C). The collected supernatant corresponds to the soluble fraction. All the above buffers were supplemented with protease (Complete™ ULTRA, Roche) and phosphatase inhibitors (PhosSTOP™, Roche). Endogenous immunoprecipitations of MXRA8 were carried out for 2 h at 4 °C using an anti-MXRA8 antibody (Abcam) (Table 4) mixed with Protein A/G Magnetic Beads (Pierce) at 4 °C for 2 h. Beads were extensively washed in lysis buffer and an elution was carried out with elution buffer (70 mM TRIS HCl, pH = 7.5, 2% SDS, 0.5 mM EDTA, and 350 mM 2-mercaptoethanol).

Tryptic digestion of the samples was performed using S-Trap Micro Spin Columns (ProtiFi, USA) according to the manufacturer’s instructions. Briefly, samples were acidified by adding phosphoric acid to a final concentration of 1.2%, seven volumes of binding buffer (90% MeOH, 100 mM TEAB, pH = 7.1) were added to the samples, which were then transferred to the S-Traps, and spun at 4000 *g* for 30 s. The trapped proteins were washed three times with the binding buffer. Protein digestion was performed by adding 2 µg trypsin (Pierce, Thermo Scientific 90057) in 125 µl of 50 mM TEAB and incubating for 2 h at 47 °C according to the manufacturer’s recommendation. Peptides were eluted with 0.2% FA and 0.2% FA/50% ACN and dried using vacuum centrifugation. Next, samples were resuspended in 40 μl of 0.1% FA and 10 μl was loaded onto disposable LC trap tips (Evotip).

NanoLC-MS/MS analysis was performed on an Evosep One UPLC system (Evosep) coupled to an Orbitrap Fusion Lumos Tribrid mass spectrometer equipped with a field asymmetric ion mobility spectrometry (FAIMS) pro ion mobility device (both from Thermo Fisher Scientific). Peptides were separated on an Endurance 30SPD (Evosep) C18 analytical column (1.9 µm, 150 µm i.d. × 15 cm) using the 44-min “30 sample per day” nonlinear gradient of Evosep One. Solvent A consisted of 0.1% FA, while solvent B contained 0.1% FA in ACN. The column temperature was 30 °C. A DDA method was applied, where MS1 full scans were acquired with a resolution of 60,000 (at 200 *m/z*), applying custom normalized target AGC values of 50%, and a maximum injection time of 250 ms, using a mass range of *m/z* 300–1500. The most intense multiply charged ions (*z* = 2–5) were fragmented using HCD activation, applying stepped collision energies of NCE = 32 and 35. MS2 scans were acquired in the linear ion trap with a rapid scan rate, applying the standard AGC target and a maximum IT of 100 ms. The ion selection threshold was set to 1 ×10^4^ and the dynamic exclusion to 60 s. Data were acquired in 1.5 s cycles applying alternating FAIMS CV voltages of −70 or −50 V.

Protein identification and label-free quantification was performed using Proteome Discoverer v3.0 SP1 (Thermo Fisher Scientific) with SEQUEST HT as the search engine and a human protein database, downloaded from SwissProt on 13 September 2023, concatenated with a “common contaminants” database. Trypsin was specified as the protease, allowing for up to one missed cleavage. The mass tolerance was set to 5 ppm for MS1 and 0.6 Da for MS2. Methylthio modification of cysteine was set as a static modification, while methionine oxidation, pyroglutamine formation of peptide N-terminal glutamine, and protein N-terminal acetylation were included as dynamic modifications. Peptides and corresponding proteins were identified at a 1% FDR. High-confidence peptide identifications of Sequest XCorr scores ≥ 1 were used to calculate abundance ratios across the sample groups using the “unique + razor” approach. Peptide abundances were normalized using the total peptide amount approach. Protein ratio calculations were pairwise ratio-based, and the hypothesis test was a background-based *t*-test. Expression levels of |log_2_FC| ≥ 1 with adjusted *p* values ≤ 0.05 were accepted as significant.

Protein abundance data from the co-immunoprecipitation experiment was processed using R v4.3.1. All proteins identified with “high” confidence, i.e., with an FDR < 0.05 and not classified as contaminants, were filtered. Further, given that the iN samples were not pure cultures and still retained a large proportion of fibroblasts, all proteins identified as differentially abundant in the anti-IgG immunoprecipitation of Ctrl fibroblasts when compared with that of Ctrl-iNs (Supplementary Table 14), were further excluded as background. In addition, only proteins quantified in at least three samples belonging to one group and with an adjusted *p*-value < 0.05 in the pairwise ratio test were considered significant.

### Statistical analysis

In the iN morphology and MXRA8 spot analyses, each dot represents an HD or Ctrl donor cell line. Each of these values are an average of several individual cells from at least two individual wells (technical duplicates). The average relative value of a donor cell line was defined as the average value of all wells per line divided by the average value of all control wells. The acquisition and analysis of all wells was performed with identical settings of the HCS microscopy. All represented neuronal profiling values of a donor cell line (cell body area, neurite count, width, length, area, and branchpoint count) are average relative values of several individual cells from at least two individual wells. The average relative dot number and area values of a donor cell line in autophagy analysis was defined as the average dot number or area of all wells per donor cell line divided by the average dot number and area of all control wells. The acquisition and analysis of all wells was performed with identical settings of the HCS microscopy.

To test differences between the two groups two-tailed unpaired or paired *t*-tests were used. For comparison of more than two groups, one-way ANOVA or nonparametric Kruskal–Wallis tests were used, according to the testing of the distribution of the data by Shaphiro–Wilk or D’Agostino–Pearson omnibus normality tests. Data are presented as dots and means, with error bars representing standard error of the mean (SEM), as specified in each figure legend.

## Results

### HD-iNs display a distinct phosphoproteomic profile

**Fig. 1:**
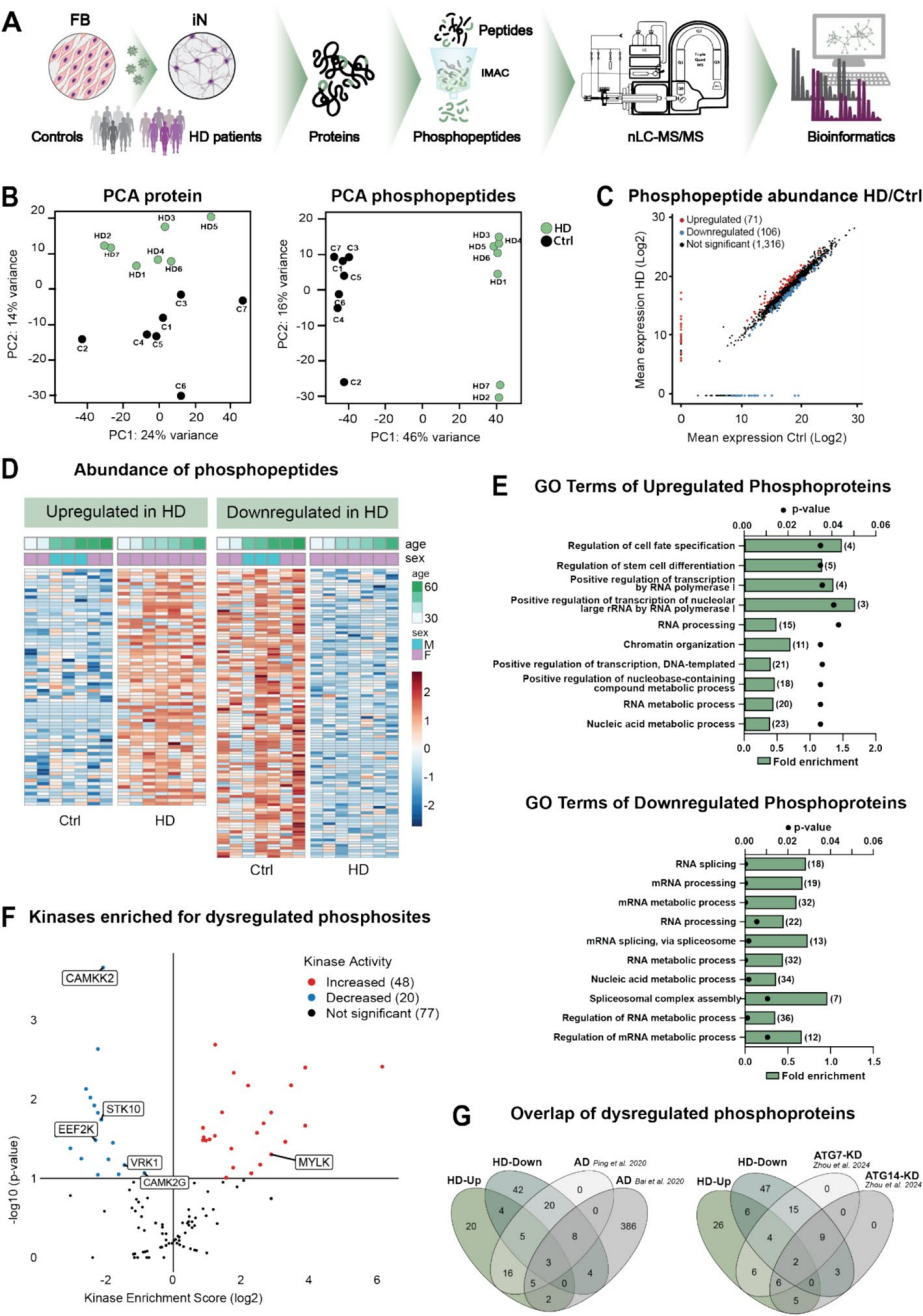
HD-iNs show a distinct phosphoproteomic profile. **a** Schematic overview of the experimental procedure. **b** PCA plots of nanoLC/MS and phospho-nanoLC/MS data of iN samples grouped by control (Ctrl) (*n* = 7) and HD (*n* = 7). **c** Scatter plot displaying log_2_ means of abundance of phosphopeptides in iNs from Ctrl (*x*-axis) and HD (*y*-axis) samples. Phosphopeptides significantly upregulated in HD-iNs when compared with Ctrl-iNs are represented by red dots, while those that are significantly downregulated are represented by blue dots, and the nonsignificant ones by black dots (Shapiro–Wilk test *p* > 0.05; unpaired *t*-test *p* < 0.05). **d** Heat maps showing the abundance of upregulated phosphopeptides (left) and downregulated phosphopeptides (right) in each sample. **e** Gene Ontology over-representation testing (using STRING-db v12.0) of genes associated with the upregulated phosphopeptides (top) and downregulated phosphopeptides (bottom). The top ten most significant terms are shown. Bar plots represent fold enrichment (strength), dots represent Benjamini–Hochberg false discovery rates (*n* = 7 control and 7 HD-iN lines, FDR < 0.05). **f** Volcano plot showing the predicted kinases enriched for the phosphosites associated with the differentially abundant phosphopeptides. Kinases predicted to have an increased activity are shown in red, while those with decreased activity are shown in blue. Among other factors, significance was measured at *p* < 0.05. **g** Venn diagrams showing the number of intersections of proteins associated with upregulated and downregulated phosphopeptides in the HD-iNs with those in (left) autophagy disruption experiments in hiPSC-iNs, and (right) studies of Alzheimer’s disease in post mortem frontal cortex samples.

We performed mass spectrometry-based phosphoproteomics (P-MS) on neuronally enriched cultures (hereafter referred to as iNs) derived from fibroblasts of seven individuals diagnosed with HD and seven age-matched controls (Fig. 1a; Table 1). These iNs, previously characterized in our 2022 study [22], were generated using a well-established protocol [21, 24, 25, 38]. Our earlier findings showed that iNs from HD donors exhibit age-related epigenetic signatures, distinct proteomic profiles, and impaired autophagy [22].

We identified a total of 5658 phosphopeptides and 4866 assigned phosphosites. The characteristics of these phosphopeptides, including their detection frequency across samples and distribution of phosphosites per peptide, are summarized in Supplementary Fig. 1a and b. After filtering for phosphopeptides present in at least four samples and correcting for the intensities on the basis of matching proteomic data (Methods), 4183 phosphopeptides corresponding to 1409 unique phosphoproteins were retained for downstream analysis (Supplementary Table 1).

Principal component analysis (PCA) revealed greater separation between HD-iNs and Ctrl-iNs in the phosphoproteomic data compared with the proteomic profiles of the same samples presented in Pircs et al. [22], suggesting more pronounced post-translational differences in HD (Fig. 1b). Differential abundance analysis identified 177 significantly altered phosphopeptides corresponding to 129 proteins. Among these, 71 phosphopeptides (from 55 proteins) were upregulated, and 106 phosphopeptides (from 86 proteins) were downregulated in HD-iNs compared with Ctrl-iNs (Fig. 1c, d; Supplementary Tables 2 and 3). Notably, 12 phosphoproteins had both upregulated and downregulated phosphopeptides. While we detected phosphopeptides from HTT (specifically Ser417, Ser419, and Ser432), which have previously been linked to disease pathology [39–41], their abundance was not significantly different between HD-iNs and Ctrl-iNs.

Interestingly, comparison with proteomic and transcriptomic datasets from Pircs et al. [22] revealed limited overlap with the phosphoproteomic changes (Supplementary Fig. 1c–f). Out of 129 genes corresponding to differentially abundant phosphopeptides, only a small subset showed altered expression at other molecular levels. Eight genes with downregulated phosphopeptides were upregulated at the proteomic level in HD-iNs, namely, AKAP8, ARHGEF6, FTO, MXRA8, NFXL1, SF3A3, TMEM214, and ZNF22 (Supplementary Fig. 1c and d). Among these, several—AKAP8, ARHGEF6, FTO, NFXL1, SF3A3, and ZNF22—are involved in transcriptional regulation, RNA processing, or neuronal signaling [42–51]. This suggests dysregulation of nuclear and synaptic pathways at the post-translational level, with the increased protein abundance potentially reflecting a compensatory response to impaired post-translational control mechanisms in HD. In addition, five genes, namely, *AKAP8*, *NFXL1*, and *STRIP1* with downregulated phosphopeptides, and *TCOF1* and *ST5*, with upregulated phosphopeptides, were upregulated at the transcriptomic level (Supplementary Fig. 1e and f). Notably, only *AKAP8* and *NFXL1* were dysregulated across all three expression levels: transcriptomic, proteomic, and phosphoproteomic (Supplementary Fig. 1d and f). AKAP8 and NFXL1 are both involved in RNA processing and transcriptional regulation [46, 47, 49], suggesting they may play key roles in mediating HD-related changes at the post-transcriptional level.

Furthermore, the presence of serine/arginine repetitive matrix proteins (SRRM1 and SRRM2) in human HD-iNs, combined with the identification of phosphoproteomic changes previously reported in their mouse homologs (SRRM1, SRRM2) as well as in CLIP2, MAP1B, PI4KB, and synaptopodin (SYNPO), suggests conserved disruption of splicing pathways [52, 53]. Together, these observations provide additional support that HD involves pronounced post-transcriptional and post-translational alterations, consistent with known splicing and RNA processing disruptions in HD [54–58].

### Functional annotation reveals dysregulated kinase activity and pathways

To better understand the functional consequences of the phosphoproteomic changes, we performed a functional enrichment analysis on the proteins associated with the dysregulated phosphopeptides, using STRING-db. Gene Ontology (GO) terms for the upregulated phosphopeptides were significantly enriched for biological processes such as cell fate specification and transcriptional regulation, while those for the downregulated phosphopeptides were primarily associated with RNA splicing (Fig. 1e; Supplementary Tables 4 and 5). Reactome pathway terms showed an enrichment for autophagy-related pathways, including PTEN regulation and PIP3-AKT signaling, for the upregulated phosphopeptides (Supplementary Fig. 1g and h; Supplementary Tables 6 and 7), suggesting a potential post-translational contribution to autophagy dysregulation in HD-iNs.

We next performed kinase enrichment analysis using the Kinase Library’s PSEA module on PhosphoSitePlus [32–34] to identify potential kinases responsible for the observed phosphopeptide changes. Among the predicted kinases overlapping with those previously quantified in the proteomic dataset [22], we identified 48 with increased activity and 20 with decreased activity in the HD-iNs (Fig. 1f; Supplementary Table 8). The kinases with increased activity predominantly belonged to the calcium/calmodulin-dependent protein kinase (CAMK) and cAMP-dependent protein kinase (PKA), cGMP-dependent kinase (PKG), and protein kinase C (PKC) families, while those with decreased activity were largely from the STE serine/threonine kinase family (Supplementary Fig. 2a).

Of the kinases previously found to be significantly differentially abundant at the proteomic level in the HD-iNs, only four showed both predicted kinase activity and changes in protein abundance in the same direction. These included CAMK2G, CAMKK2, EEF2K, and STK10, all with decreased predicted activity and downregulation in the HD-iNs (Fig. 1f; Supplementary Fig. 2b; Supplementary Table 8; uncropped WB images in the Supplementary Information file). Notably, these proteins did not differ significantly in the proteomic data from the parental fibroblasts, suggesting that the differences were specific to the iNs (Supplementary Fig. 2c). The remaining predicted kinases that were differentially abundant—MYLK and VRK1—did not show consistent trends between predicted activity and protein levels (Supplementary Table 8).

Together, these findings highlight widespread alterations in signaling and phosphorylation regulation in HD-iNs, driven in part by dysregulated kinase activity across multiple functional families. Notably, many of the kinases with altered activity are linked to autophagy-related pathways, further supporting the concept that post-translational dysregulation of autophagy is a central feature of HD-iNs. Although our findings associate MXRA8 Ser377 phosphorylation with autophagy-related pathways at the network level, functional validation of autophagy regulation by pSer377-MXRA8 will require phospho-selective imaging and/or targeted genetic perturbations.

### Shared phosphoproteomic signatures with autophagy disruption and neurodegeneration

To determine whether the phosphoproteomic alterations observed in HD-iNs are shared with other models of neurodegeneration, we compared our dataset with phosphoproteomic profiles from three previously published sources.

First, we found substantial overlap with phosphoproteomic changes observed in autophagy-disrupted human iPSC-derived neurons with CRISPRi-mediated knockdown of *ATG7* and *ATG14*, as described by Zhou et al., 2024 [59] (Supplementary Table 9). Specifically, 27 phosphoproteins with downregulated phosphopeptides and 17 with upregulated phosphopeptides in HD-iNs were also dysregulated in these autophagy-deficient models (Fig. 1g; Supplementary Fig. 2d; Supplementary Table 9). This suggests that phosphoproteins altered in HD-iNs may contribute to, or be influenced by, autophagy dysfunction.

Second, we observed that 32 phosphoproteins with downregulated phosphopeptides and 23 with upregulated phosphopeptides in HD-iNs overlapped with phosphoproteomic alterations in post mortem frontal cortices of patients with Alzheimer’s disease (AD) [60, 61] (Fig. 1g; Supplementary Fig. 2d; Supplementary Table 10). This suggests that similar signaling pathways may be disrupted across different age-related neurodegenerative diseases, highlighting the broader importance of post-translational dysregulation in these conditions.

### ON–OFF and OFF–ON phosphoproteins reveal binary phospho-switches in HD-iNs

**Fig. 2:**
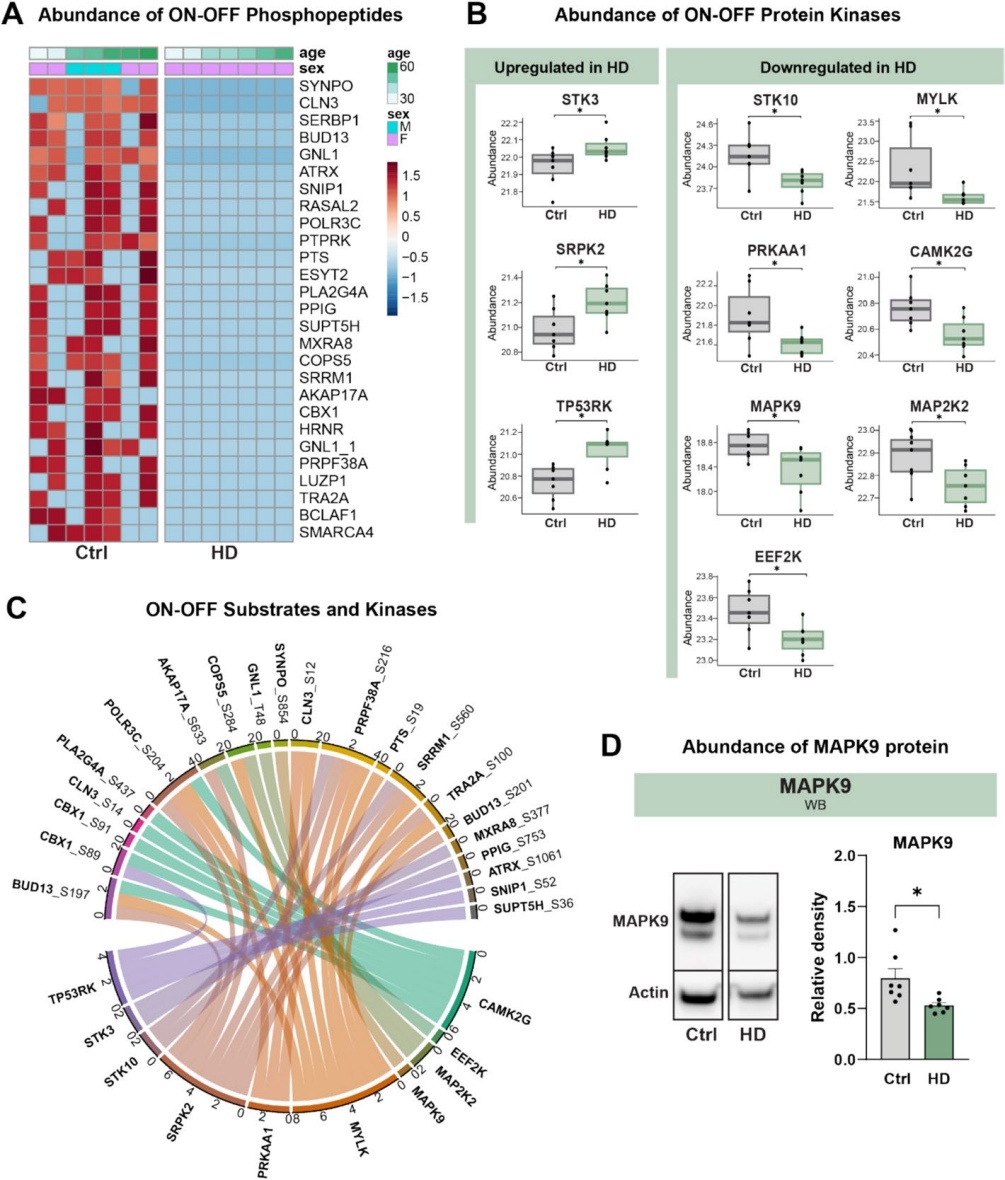
ON–OFF proteins and their predicted kinases show significantly altered abundance in HD-iNs. **a** Heat map showing the abundance of the ON–OFF phosphopeptides in each sample. **b** Box plots showing the abundance of the kinases, predicted to phosphorylate ON–OFF phosphosites, that are upregulated in HD-iNs when compared with Ctrl-iNs (left), or downregulated in HD-iNs (right). (*n* = 7 control and 7 HD iN donor cell lines). **c** Chord diagram summarizing the interactions between the predicted kinases and the corresponding ON–OFF phosphosites. The scales represent the number of connections. **d** Validation of MAPK9 protein abundance by WB of seven Ctrl and seven HD-iNs. **b**, **d** Each dot represents one Ctrl or one HD adult human donor cell line from the converted iNs. For statistical analysis two-tailed *t*-tests were used in all cases. *p < 0.05. Data are shown as mean ± SEM in **d.**

We identified a subset of 27 downregulated phosphopeptides, corresponding to 26 phosphoproteins, that were entirely absent in HD-iNs but present in at least four Ctrl-iNs (Fig. 2a; Supplementary Table 11). We refer to these as ON–OFF phosphopeptides/proteins.

**Fig. 3:**
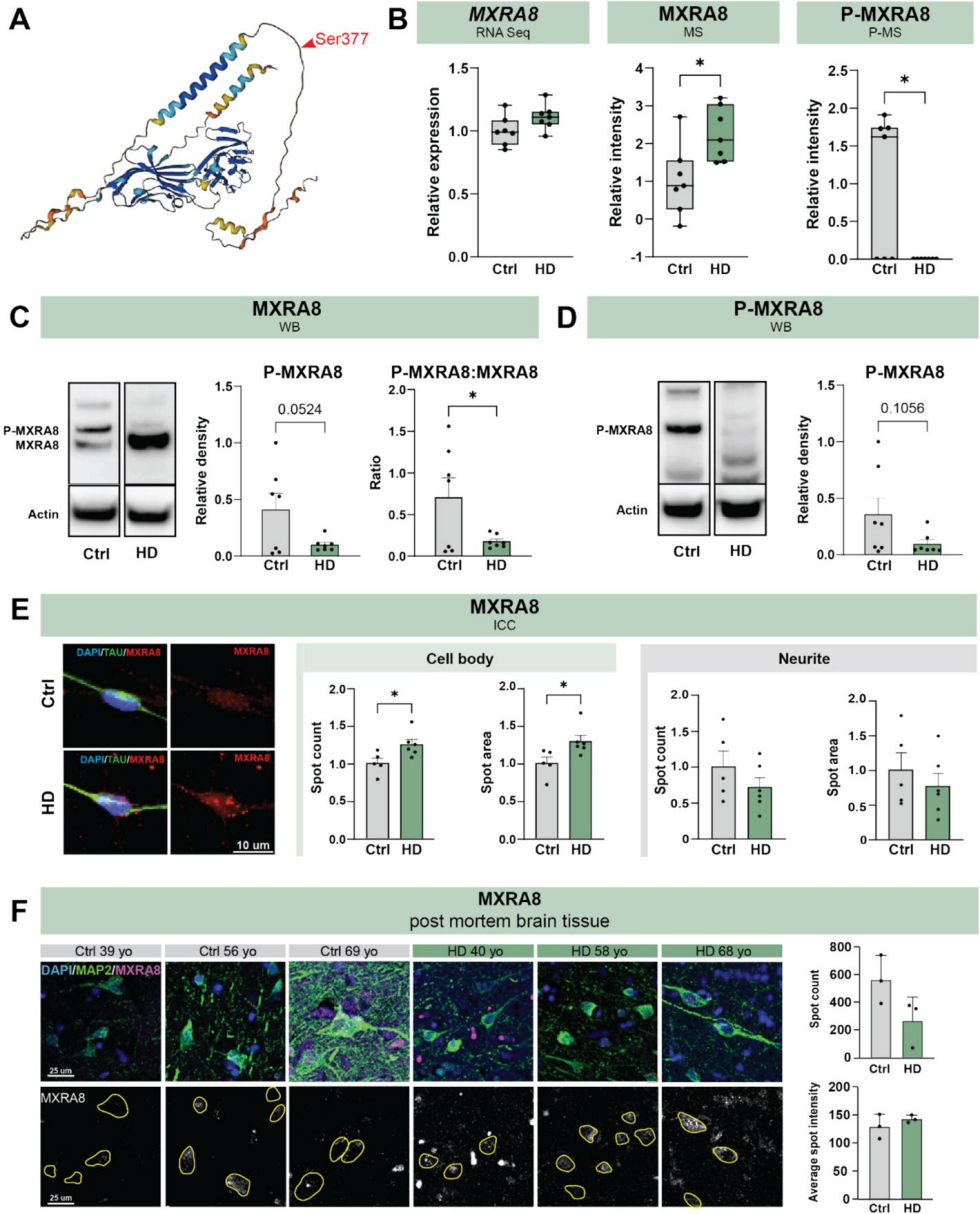
HD-iNs show alteration of abundance, phosphorylation, and localization of MXRA8 protein. **a** Three-dimensional (3D) structural image of the MXRA8 protein from AlphaFold. The red arrow points to the specific phosphosite, Ser377, which is completely diminished in HD-iNs. **b** RNA expression, protein abundance, and phosphoprotein abundance of MXRA8 in seven Ctrl- and seven HD-iNs from RNA sequencing, MS, and P-MS analyses, respectively. **c** WB validation of MXRA8 protein abundance of *n* = 13 of *N* = 7 Ctrl-iNs (two biological replicates per each line, except for C2) and *n* = 14 of *N* = 7 HD-iNs (two biological replicates per each line) using native anti-MXRA8 (“*N*” represents the number of donor cell lines, “*n*” represents the total number of biological replicates). Blot sections show one representative Ctrl-iN and HD-iN sample. Column diagrams show the relative density of P-MXRA8 and a ratio of P-MXRA8:MXRA8 as analyzed on the native MXRA8-labeled blots. **d** WB validation of P-MXRA8 protein abundance of *n* = 14 of *N* = 7 Ctrl and *n* = 14 of *N* = 7 HD iNs using a custom designed phospho-Ser377-MXRA8 specific antibody. Blot sections show one representative Ctrl- and HD-iN sample. Column diagrams show the relative density of P-MXRA8. **e** Validation of MXRA8 protein abundance and localization by HCS in TAU-counterstained samples (seven Ctrl- and seven HD-iN samples). Bars show the spot count and area in the cell body (left) and in the neurites (right) of Ctrl- and HD-iNs. Representative images show one Ctrl- and one HD-iN sample stained with DAPI, TAU, and MXRA8. **f** IHC validation of MXRA8 protein abundance in MAP2- and DAPI-counterstained samples: three Ctrl- and three HD post mortem human cortical brain samples. Bars show the spot count and average spot intensity in the cell body. On representative images, yellow outlines mark neuronal cell bodies on the basis of MAP2 staining. Scale bar 25 μm. (*n* = 3 of *N* = 3 HD; *n* = 3 of *N* = 3 Ctrl, where *N* represents the number of post mortem brain samples and *n* represents the total number of biological replicates). **b**–**e** Each dot represents one Ctrl- or one HD- adult human cell line from the converted iNs, the columns of the bars show the mean value of the individual values, the error bar indicates SEM. For statistical analysis, two-tailed *t*-tests were used, *p < 0.05.

Except for MXRA8, which was upregulated at the proteomic level (Fig. 3b), none of the ON–OFF proteins showed significant changes at either the transcriptomic or proteomic levels (Supplementary Fig. 3a and b; Supplementary Table 11). Notably, two ON–OFF phosphoproteins—SRRM1 and SYNPO—have previously been reported to be dysregulated in the striatal and hippocampal phosphoproteomes of HD mouse models [12, 13]. Furthermore, SRRM1, along with nine additional ON–OFF phosphoproteins (BCLAF1, CLN3, ESYT2, PPIG, PRPF38A, SERBP1, SMARCA4, SNIP1, and SUPT5H), were also found to be dysregulated in autophagy-deficient iPSC-derived neurons from *ATG7* and *ATG14* knockdown experiments [59] (Supplementary Fig. 3f; Supplementary Table 9). In addition, BCLAF1, SMARCA4, SRRM1, and SYNPO were also identified to be dysregulated in the post mortem frontal cortex phosphoproteomes of patients with AD [60, 61] (Supplementary Fig. 3f; Supplementary Table 10). Among these, SRRM1 is of particular interest, as it is a core component of the splicing machinery. Its phospho-dysregulation links RNA processing to both autophagy impairment and age-related neurodegeneration and highlights the intersection of post-transcriptional regulation with disease-relevant cellular pathways [62, 63].

We additionally identified a subset of 19 OFF–ON phosphopeptides, corresponding to 17 phosphoproteins, which were completely absent in Ctrl-iNs but expressed in at least four HD-iN samples (Supplementary Fig. 3c; Supplementary Table 12). None of the genes corresponding to these phosphopeptides were dysregulated at other levels of expression in the HD-iNs, except for treacle ribosome biogenesis factor 1 (TCOF1), which was upregulated at the transcriptomic level (Supplementary Fig. 3d and e). Among these, CLIP2 (CAP-Gly domain-containing linker protein 2) was previously reported as dysregulated in the phosphoproteome of HD mouse models [12, 13]. In addition, TCOF1 and CLIP2—as well as IWS1, NCL, NDRG2, and UBE2O—were also found to be altered in the phosphoproteomes of autophagy-disrupted iPSC-derived neurons and post mortem frontal cortices from patients with AD (Supplementary Fig. 3f; Supplementary Tables 9 and 10) [59–61].

Together, the ON–OFF and OFF–ON phosphoproteins represent strong, binary phospho-switches in HD-iNs, marked by either a complete presence or complete absence of phosphorylation. The fact that many of these proteins are also found in HD models, autophagy-impaired neurons, and AD brain tissue suggests they may play important roles in regulating cell signaling and stress responses in neurodegenerative conditions.

### Dysregulated ON–OFF kinases link phospho-switches to autophagy and neurodegenerative signaling

Using the kinase prediction tool from the PhosphoSitePlus database [32–34], we identified potential upstream kinases for the ON–OFF phosphosites (Supplementary Table 13). Overall, we considered kinases that were expressed in the global proteomic dataset and had a site percentile greater than 90 in the iNs, as potential kinases. Further, among those, ten kinases were differentially abundant in the global proteomic data: SRPK2, STK3, and TP53RK were upregulated in the HD-iNs, while CAMK2G, EEF2K, MAP2K2, MAPK9, MYLK, PRKAA1, and STK10 were downregulated (Fig. 2b). Except for SRPK2 and TP53RK, none of the other listed candidate kinases showed significantly altered abundances in the corresponding parental fibroblasts, suggesting that these differences are not simply shared with the parental fibroblast state and may reflect context-dependent changes in the converted cultures (Supplementary Figs. 2C and 3G). Several of these differentially abundant kinases were linked to multiple ON–OFF phosphosites, highlighting their potential regulatory significance.

Notably, PRKAA1 (AMPKα1), a central kinase in the CAMKK–AMPK autophagy pathway, along with MYLK and CAMK2G, was predicted to phosphorylate CLN3, a core autophagy-associated protein [64], at serine 12 (S12) and CAMK2G at S14 (Fig. 2c). These observations suggest that reduced levels of PRKAA1, MYLK, or CAMK2G may underlie the lack of CLN3 phosphorylation in HD-iNs. Importantly, this links the dysregulation of autophagy-related signaling directly to the ON–OFF phosphoproteome.

While some of the ON–OFF phosphosites had multiple predicted kinases, two showed unique predicted kinase–substrate relationships: SYNPO-S854, phosphorylated by MAPK9, and AKAP17A-S633, by EEF2K (Fig. 2c). MAPK9, also known as JNK2 (c-Jun N-terminal kinase 2), is a stress-activated kinase with established roles in oxidative stress responses, apoptosis, and neurodegeneration [65–67]. WB analysis confirmed reduced MAPK9 protein levels in HD-iNs compared with Ctrl-iNs, consistent with our proteomic results (Fig. 2d).

SYNPO, the substrate of MAPK9 at S854, is a cytoskeletal-associated protein highly enriched in neurons [68–72]. It plays a critical role in dendritic spine structure, actin organization, and synaptic plasticity—all processes vulnerable in neurodegenerative diseases [68, 73–75]. Its absence of phosphorylation in HD-iNs, paired with reduced MAPK9, may reflect impaired structural and signaling integrity in disease-relevant neuronal compartments.

In summary, kinase prediction analysis of ON–OFF phosphoproteins uncovered multiple potential regulators with strong connections to autophagy and neurodegenerative pathways. Dysregulation of key kinases such as MAPK9, PRKAA1, MYLK, and CAMK2G, and their links to substrates such as CLN3 and SYNPO, support the hypothesis that the loss of site-specific phosphorylation contributes to impaired signaling in HD-iNs, with broader implications for cellular stress responses and synaptic function.

### Matrix-remodeling protein 8 shows distinct alterations in HD-iNs

We selected MXRA8 for further investigation, as it was the only ON–OFF phosphoprotein that also showed a significant change in overall protein abundance in HD-iNs (Fig. 3a, b). Notably, MXRA8 abundance did not differ significantly in the corresponding parental fibroblasts, suggesting that the observed differences may be specific to the iNs (Supplementary Fig. 4a). Although the phospho-Ser377 peptide of MXRA8 was completely absent in HD-iNs, the overall protein levels of MXRA8 were increased, as detected by conventional MS (Fig. 3b). The observed increased expression of the MXRA8 protein could potentially be a compensatory response to the loss of phosphorylated MXRA8. Given that the RNA sequencing data revealed no significant difference in *MXRA8* mRNA expression, the observed differences likely arise from post-transcriptional regulation (Fig. 3b). Notably, MXRA8 had only one phosphopeptide with a single phosphosite detected across the entire dataset, making it uniquely suited for targeted follow-up studies. We hypothesize that the combination of phosphorylation loss and protein-level upregulation may reflect a dysregulated state of MXRA8 in the HD-iNs, pointing to a possible role in disease-related mechanisms.

MXRA8—also known as limitrin, dual immunoglobulin domain-containing cell adhesion molecule (DICAM), or adipocyte-specific protein 3 (ASP3)—is a transmembrane protein belonging to the immunoglobulin superfamily [26]. It is involved in various cellular processes, including mesenchymal cell differentiation, osteoclast development, angiogenesis, and viral entry [26, 76–78]. In the central nervous system, MXRA8 has been primarily described in glial cells, where it contributes to blood–brain barrier integrity and modulates astrocyte-mediated neuroinflammation [26, 79]. However, its role in neurons remains largely unknown. Our findings—showing a strong imbalance between MXRA8 phosphorylation and protein abundance in HD-iNs—suggest a previously unrecognized role for MXRA8 in neurons, and suggest its possible involvement in neurodegenerative processes.

Therefore, we first validated the MS and P-MS data by measuring the abundance and ratio of MXRA8 and P-MXRA8 by WB using either a native anti-MXRA8 antibody (labeling both native and post-translationally modified forms of MXRA8) or our custom-designed phospho-Ser377-MXRA8 specific antibody (Fig. 3c, d; Supplementary Fig. 4b; uncropped WB images in the Supplementary Information file). On the basis of the evaluation of the native anti-MXRA8-labeled blots, HD-iNs showed a lower abundance of P-MXRA8 compared with Ctrl-iNs, the difference of which was close to significance (*p* = 0.0524). By contrast, the ratio of P-MXRA8:MXRA8 protein abundance showed a significant decrease in HD-iNs compared with nondiseased controls. The P-MXRA8 detection using the phospho-Ser377-MXRA8 specific antibody also confirmed a decreased abundance of P-MXRA8 in HD-iNs seen by the native anti-MXRA8 antibody (Fig. 3d). The lack of statistical significance for total MXRA8 in WB likely reflects the lower sensitivity of bulk WB compared with MS or ratio analyses for subtle changes.

Next, we performed ICC staining followed by high-content microscopy to visualize MXRA8, using the native anti-MXRA8 antibody and counterstaining with TAU (Fig. 3e; Supplementary Fig. 4c). MXRA8 staining appeared as punctate signals, primarily localized in the cell body and nuclei but was also present in the neurites of iNs (Fig. 3e; Supplementary Fig. 4c). In HD-iNs, the size and number of MXRA8-positive puncta were significantly increased in the cell bodies. In contrast, the neurites showed a trend toward fewer MXRA8 puncta, although this difference did not reach statistical significance (Fig. 3e). To further assess the neuronal relevance of these findings, we performed IHC in post mortem cortical tissue from three patients with advanced-stage (grade 4) HD and three age-matched controls, using MAP2 as a neuronal marker alongside MXRA8 (Fig. 3f). Similar to the HD-iNs, we observed MAP2-positive neurons with dense punctate MXRA8 staining in HD patient tissue. However, given the limited tissue availability, small sample size, and heterogeneity between donors, the analysis lacked sufficient statistical power to support a robust quantitative assessment. Overall, these observations provide supportive evidence that MXRA8 is expressed as puncta in human cortical neurons, including in HD brain tissue.

These findings reveal a marked imbalance between MXRA8 phosphorylation and protein abundance in HD-iNs, accompanied by distinct subcellular redistribution. In addition, MXRA8 punctate staining was observed in human HD cortical neurons, supporting the relevance of further investigating the role of MXRA8 in neurons in the context of HD.

### MXRA8-associated interactome and kinases suggest a role in autophagy and neurovascular function

**Fig. 4:**
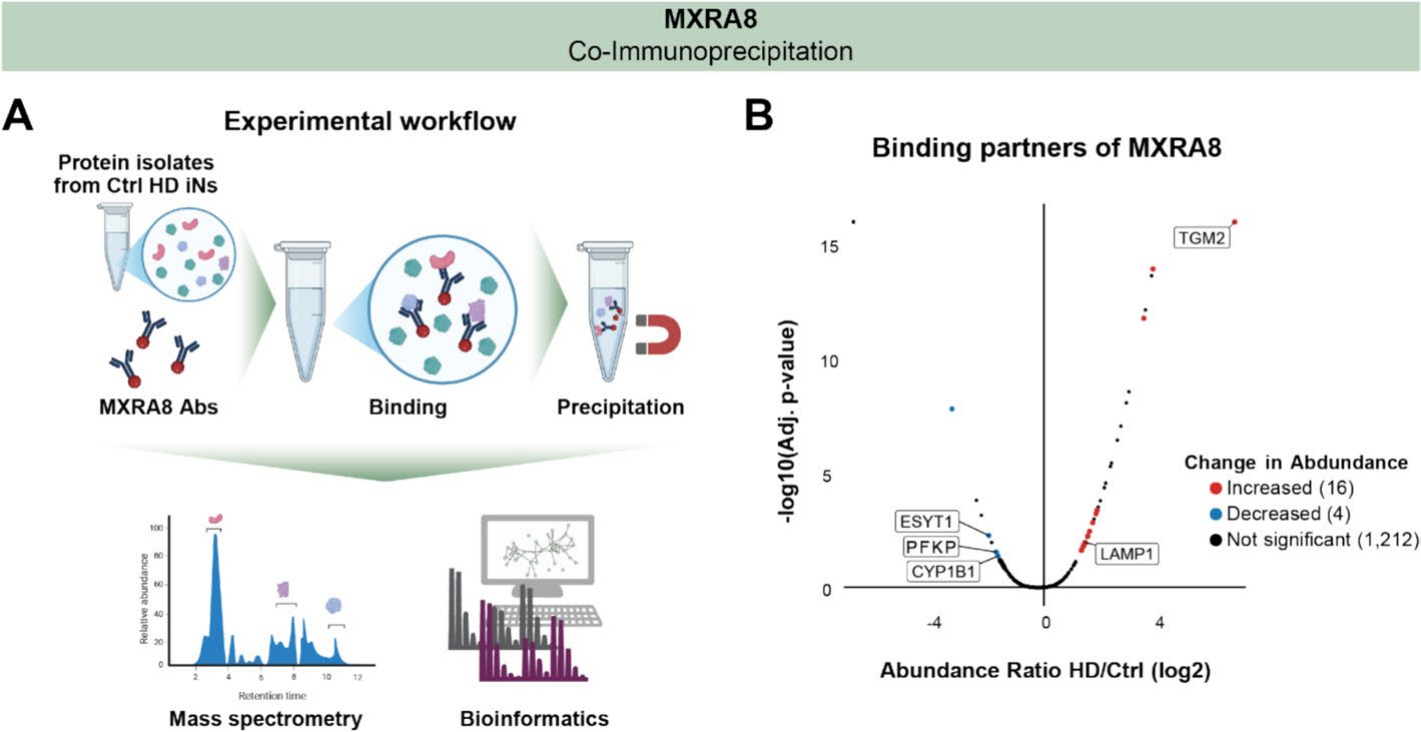
Co-immunoprecipitation identifies differences in the profile of binding partners of MXRA8 in HD-iNs. **a** Schematic overview of the co-immunoprecipitation experimental procedure. **b** Volcano plot of identified binding partners of MXRA8 of *n* = 4 Ctrl- and *n* = 4 HD-iN samples. On the volcano plot, binding partners with increased abundance in HD-iNs are shown in red, while those with decreased abundance are shown in blue, and the nonsignificant ones in black dots. Significance was measured on the basis of an adjusted *p*-value < 0.05 among other parameters.

To explore the molecular context of MXRA8 and gain insight into its potential functions in neurons, we performed co-IP using the native anti-MXRA8 antibody, followed by LC–MS/MS, on four control and four HD-iNs (Fig. 4a; Supplementary Table 15). Overall, we identified 1232 proteins with high confidence (FDR < 0.05; Fig. 4b). From these, we shortlisted 20 candidate interactors on the basis of significant abundance ratio and reliable peptide-level quantification in at least three samples within one condition (Fig. 4b; Supplementary Table 16). Of these, five proteins were robustly represented in ≥ 50% of samples in either group (Fig. 4b).

Among the candidates, transglutaminase 2 (TGM2) and lysosomal-associated membrane protein 1 (LAMP1) showed increased binding to MXRA8 in HD-iNs, while cytochrome P450 family 1 subfamily B member 1 (CYP1B1), phosphofructokinase (PFKP), and extended synaptogamin 1 (ESYT1) were decreased compared with controls (Fig. 4b; Supplementary Table 16). TGM2 is known to modulate neurogenesis and synaptic pruning as well as mediating autophagosome–lysosome fusion, while LAMP1 is a key component of macroautophagy [80–85]. CYP1B1 is involved in blood–brain barrier integrity and inflammatory responses, PFKP is a critical glycolytic enzyme, and ESYT1 regulates calcium transport at membrane contact sites [86–89]. These changes in binding profiles suggest a broader role for MXRA8 in maintaining neuronal homeostasis and metabolic balance.

Owing to the inability to perform co-immunoprecipitation with our custom-made anti-MXRA8-pS377 antibody and the lack of experimental studies on MXRA8 phosphosites, the kinases regulating its phosphorylation remain unidentified, we performed prediction analysis on the kinase interactome profile of MXRA8-pS377 Kinase Library’s kinase prediction webtool. Among the list of kinases of MXRA8-pS377 that we had predicted earlier with a site percentile greater than 90 and expressed in the global proteomic data (Supplementary Table 13), AAK1, BMP2K, DSTYK, EIF2AK2, GAK, IRAK4, PRKCA, PRKCD, PRKCE, and STK26 had the highest site scores (Supplementary Fig. 4d). The site score reflects the similarity between the phosphosite and a given kinase’s preferred consensus motif, making it an estimate of how likely the kinase is to phosphorylate the phosphosite. Further experiments with S377A/S377D mutant interactomes would need to be performed to further validate the role of these kinases in the phosphorylation of MXRA8-pS377.

Together, these findings suggest that altered kinase activity and changes in MXRA8 protein–protein interactions may influence its phosphorylation state and function in HD-iNs. The association of MXRA8 with proteins involved in metabolism, calcium signaling, and the lysosomal/autophagy axis, along with potential regulation by autophagy-linked kinases, points to a broader role in neurovascular integrity and stress response pathways relevant to neurodegeneration.

## Discussion

Although the causative genetic mutation was recognized over 30 years ago, the exact pathogenesis of this condition remains unknown [2]. Epigenetic hallmarks and neuronal age are both key to HD pathology, as symptoms occur only after a certain age despite the mutation being inherited [19]. In this study we used our previously published proteomic and transcriptomic dataset of neuronally enriched HD-iN cultures and expanded it with P-MS measurements to investigate the phosphoproteomic profiles of patient-derived directly reprogrammed HD-iNs.

Importantly, phosphoproteomics provides a dynamic view of protein regulation that is not captured by steady-state transcriptomic or proteomic analyses, allowing us to study key signaling disruptions that may precede overt disease phenotypes. HD-iNs not only retain the patient genetic mutation but also recapitulate the epigenetic age of the donor [19, 22, 24]. We previously found that HD-iNs (*i*) mainly differ on a proteomic, but not so much on a transcriptomic level; (*ii*) show a less elaborate neuronal morphology; (*iii*) show an accelerated epigenetic aging on the basis of DNA methylation arrays; and (*iv*) have clear autophagy dysfunction [22].

While growing knowledge is available about the DNA methylation, transcriptomic, and proteomic profiles of HD, much less is known about the contribution of post-translational phosphorylation dysregulation to the disease mechanism [90, 91]. Previously published studies focusing on protein phosphorylation in HD mainly studied the importance of specific protein phosphorylation sites on HTT, TAU, or histone proteins [9–11, 14, 15, 92, 93]. Here we performed an untargeted phosphoproteomic profiling approach using seven HD- and seven Ctrl-iNs. This comprehensive, unbiased analysis of the phosphorylation network enabled us to identify new key proteins and dysregulated pathways, especially when combined with our previously published transcriptomic and proteomic datasets from the same patient-derived iNs and controls.

So far, such phosphoproteomic analyses have only been done in mouse models of HD. The first study published by Beaumont et al. in 2016 found no significant change in the phosphoproteome of the striatum of Q175 HD mice [94]. In 2022, Mees et al. performed a P-MS analysis of different brain regions of R6/1 transgenic mice at pre-manifest and manifest stages [12, 13]. They reported strong alterations in phosphoprotein levels in pre-manifest mice, particularly in the striatum and the cortex, but with fewer differences at manifest stages [12, 13].

The phosphoproteomic analysis presented in our study is the first phosphoproteome-wide investigation of patient-derived human samples. P-MS data showed a clear distinction between HD-iNs and Ctrl-iNs at the phosphoproteomic level. Interestingly, we only found four overlapping phosphoproteins between our HD-iN dataset and the HD mouse phosphoproteome from Mees et al. (MAP1B, PI4KB, SRRM1, and SRRM2) [12, 13]. While inconsistencies between the nomenclature of mice and human proteins may explain part of this discrepancy, the limited overlap strongly highlights the importance of studying HD in human-derived models. Mees et al. also reported that pathways such as neurite outgrowth, ubiquitination, tight junction assembly, and endocytosis were upregulated, while mRNA translation, ion homeostasis, and amino acid transport were downregulated. Cytoskeleton organization and phosphatase activity were mixed across conditions [13].

STRING-db analysis of our HD-iN P-MS data showed a downregulation of RNA splicing pathways. Out of the 18 significantly downregulated proteins related to RNA splicing, 8 were identified as ON–OFF proteins (BUD13, SNIP1, PRPF38A, TRA2A, SRRM1, PPIG, AKAP17A, and BCLAF1). Previously, we found RNA splicing to be significantly upregulated on a protein level (MS) [22]. One overlapping protein, splicing factor 3a subunit 3 (SF3A3) is present with a significantly upregulated abundance but with significantly decreased phosphorylation in HD-iNs, suggesting a possible functional disruption. At the transcriptomic level, RNA splicing-related processes were found to be significantly up- and downregulated, highlighting the complexity of post-transcriptional RNA regulation in HD-iNs [22]. These findings align with the current literature that implicates RNA splicing dysregulation as a key pathogenic mechanism in HD [54–57].

To contextualize our findings beyond HD models, we compared our dataset with other phosphoproteomic studies in human neurodegeneration, including AD and models of autophagy dysfunction. Since large-scale phosphoproteomic datasets for neurodegenerative conditions remain limited, we focused our comparisons on three high-confidence datasets: iPSC-derived neurons with CRISPRi-mediated knockdown of autophagy genes (ATG7 and ATG14) [59] and post mortem brain tissue from patients with AD [59–61]. We found significant overlap in phosphoproteomic alterations between HD-iNs and both autophagy-impaired neurons and AD brains. Specifically, 44 phosphoproteins dysregulated in HD-iNs were also affected in the autophagy-deficient neurons, and 55 overlapped with those altered in AD brains. Notably, 22 phosphoproteins were altered across all three datasets, suggesting they may act as shared molecular mediators in age- and stress-related neurodegeneration.

Notably, MAP1B, SRRM1, and SRRM2 were significantly differentially phosphorylated in the mouse HD brain as well [12, 13]. A subset of nine phosphoproteins showed conserved changes at the same phosphosite and in the same direction across these datasets, supporting the existence of conserved post-translational regulatory mechanisms. We also identified a class of ON–OFF phosphoproteins—those completely unphosphorylated in HD-iNs yet present in controls. Among these, several proteins (e.g., SRRM1, SYNPO, and BCLAF1) were also ON–OFF in both autophagy-deficient neurons and AD brains, indicating that loss of site-specific phosphorylation may represent a common binary switch mechanism in neurodegenerative pathways.

Strikingly, only one of the ON–OFF proteins, MXRA8, had a single nonphosphorylated phosphosite—at Serine-377—while having a significantly higher abundance in HD-iNs. Presumably, the diminished function of MXRA8 caused by interrupted phosphorylation resulted in a compensatory increase in protein production by the cells. Importantly, we also verified MXRA8 expression in neurons of post mortem HD cortical tissue, where punctate MXRA8 staining was observed in neuronal cell bodies. MXRA8 is a transmembrane protein of the immunoglobulin superfamily, previously characterized mainly in glial and vascular contexts, but not in neurons. While a robust assessment of MXRA8 neuronal expression was prevented by tissue availability, small sample size, and donor heterogeneity, our findings support the relevance of further exploring the impact of MXRA8 expression on human neuronal function in health and disease. Here, we demonstrate for the first time its altered phosphorylation and abundance in a neuronal context, implicating MXRA8 dysregulation in neurodegenerative processes [26, 95].

To explore the functional implications of MXRA8 dysregulation, we performed co-immunoprecipitation coupled with LC–MS/MS, identifying a selective shift in native MXRA8-associated interactions in HD-iNs. Proteins involved in lysosomal function (LAMP1) and stress homeostasis (TGM2) showed increased interaction, while partners related to glycolysis (PFKP) and calcium transport (ESYT1) displayed reduced associations in HD-iNs. These context-specific interaction shifts suggest that MXRA8 participates in metabolic and stress-response pathways disrupted in HD.

Furthermore, we predicted potential kinases regulating MXRA8-pSer377 phosphorylation. Of particular interest were AAK1, BMP2K, GAK, PRKCD, PRKCA, IRAK4, DSTYK, PRKCE, STK26, and EIF2AK2, all previously implicated in autophagy regulation [96–105]. While the current data associate MXRA8 Ser377 phosphorylation with autophagy-related networks, direct functional validation will be essential in future studies, including autophagy flux assays and compartment-specific evaluation of MXRA8 phosphorylation signaling in neurites and soma using phospho-selective imaging and/or targeted genetic perturbations with phospho-mutant MXRA8 or site-directed editing.

MXRA8 has no experimentally determined kinases, owing to a lack of studies on MXRA8 phosphosites and the overall small fraction of known kinase–substrate relationships [106–108]. This limits our ability to identify potential kinases for MXRA8 on the basis of established kinase prediction tools. Additional experimental studies that evaluate the potential of the shortlisted kinases in phosphorylating MXRA8 would therefore be required for further verification.

Taken together, our findings identify MXRA8 as a novel, neuronally relevant protein whose dysregulated phosphorylation and protein–protein interactions may contribute to autophagy impairment and neurovascular dysfunction in HD [109]. The loss of phosphorylation in combination with increased protein abundance, disrupted interactome, and reduced autophagy-linked kinase activity suggests a broader role for MXRA8 in neurodegenerative stress responses.

However, some limitations of this study warrant consideration. The reprogramming efficiency of direct neuronal transdifferentiation influences the purity and homogeneity of the resulting cell populations, with our iN model achieving approximately 25% neuronal enrichment.

Also, the directly converted iNs used here represent pan-neuronal cultures rather than a subtype-specified neuronal population [21–25]. Since this platform yields mixed pan-neuronal iN cultures, the phosphoproteomic changes identified here are interpreted at the culture level; resolving subtype-specific contributions will require targeted differentiation or cell-type-resolved approaches in future work.

A further constraint in interpreting the phosphoproteomic results stems from the paucity of phosphatase databases. Although phosphatases play critical roles in regulating protein phosphorylation, phosphatase–substrate and phosphosite annotations remain comparatively sparse and incomplete relative to kinase–substrate resources. Currently, no broadly adopted phosphosite-resolved framework enables systematic “phosphatase enrichment” analysis analogous to that available for kinases; consequently, our interpretation is biased toward kinase-centric annotations owing to these limitations in phosphatase–substrate knowledge, underscoring the need for improved phosphatase resources in future analyses.

Finally, validation in human post mortem brain tissue was constrained by sample availability, restricting inclusion to grade 4 HD cases. This end-stage representation captures only the most resilient surviving neurons, potentially confounding interpretations; if MXRA8 accumulation were pathogenic, neurons with elevated levels would likely have perished earlier and thus would not be included in our analysis. This limitation could only be overcome by including postmortem tissues from HD patients at earlier disease stages; however, such samples are limited in availability. It is also worth noting that stage-specific phosphoproteomic changes represent a promising direction for future research. Studies involving larger cohorts of donor-derived iN cultures spanning asymptomatic and symptomatic stages of HD are essential to clarify whether phosphorylation changes, including those in MXRA8, correlate with disease progression.

## Conclusions

This study presents the first phosphoproteome-wide analysis of human HD neurons, using directly reprogrammed iNs from genetically and epigenetically characterized patient donors. By integrating phosphoproteomic, proteomic, and transcriptomic data from the same individuals, we demonstrate the critical role of PTMs—especially phosphorylation—in HD pathogenesis. The divergence between phosphorylation status and gene or protein expression in many key regulators, including MXRA8, highlights that PTMs provide distinct and functionally relevant disease signals that are not captured by transcriptomic or steady-state proteomic profiling alone. Our findings not only reveal previously unrecognized HD-associated phospho-switches and disrupted signaling pathways, particularly in autophagy and stress response networks, but also identify novel candidates such as MXRA8 for mechanistic follow-up and therapeutic targeting. This multiomics approach establishes a new platform for identifying clinically relevant biomarkers and druggable targets in human neurodegeneration.

## Supplementary information

Additional file 1.

Additional file 2.

## Data Availability

All data needed to evaluate the conclusions in the paper are present in the paper and/or the Supplementary Materials section. The mass spectrometry phosphoproteomics data have been deposited to the ProteomeXchange Consortium via the PRIDE [110] partner repository with the dataset identifier PXD055857 and 10.6019/PXD055857. The mass spectrometry dataset of the co-IP experiment was uploaded to the Proteomics Identifications Database (PRIDE, [https://www.ebi.ac.uk/pride/login]). Project accession: PXD068326The code used for the data analysis and for generation of plots can be found at (https://github.com/pircs-lab/iN_HD_PMS_analysis_2025).

## Abbreviations

ACN: Acetonitrile
AD: Alzheimer’s disease
AGC: Automatic gain control
ANOVA: One-way ANOVA
ASCL1: Achaete-Scute family bHLH transcription factor 1
BRN2: POU class 3 homeobox 2, also known as POU3F2
CAMK: CAMK family
co-IP: Co-immunoprecipitation
DAPI: 4,6-Diamidino-2-phenylindole
DDA: Data dependent analysis
DMEM: Dulbecco’s modified Eagle medium
DPBS: Dulbecco’s phosphate-buffered saline
DTT: Dithiothreitol
EDTA: Ethylene diamine tetraacetic acid
EGTA: Ethylene glycol tetraacetic acid
ELISA: Enzyme-linked immunosorbent assay
FA: Formic acid
FBS: Fetal bovine serum
FDR: False discovery rate
GO: Gene Ontology
HBSS: Hanks’ balanced salt solution
HCS: High-content automated screening
HD: Huntington’s disease
HTT: Huntingtin
iNs: Induced neurons
IHC: Immunohistochemistry
IMAC: Metal ion affinity chromatography
IT: Injection time
LC–MS/MS: Liquid chromatography coupled tandem mass spectrometry
MOI: Multiplicity of infection
MS: Mass spectrometry
mHTT: Mutant HTT
MXRA8: Matrix remodeling associated protein 8
NCE: Normalized collision energy
OD: Optical density
ON–OFF: ON–OFF phosphopeptides/proteins
P-MS: Phosphoproteomic analysis by mass spectrometry
PCA: Principal component analysis
PGK: Phosphoglycerate kinase
PIC: Protease inhibitor cocktail
polyQ: Poly-glutamine
PTM: Post-translational modification
REC: Research Ethics Committee
RIPA: Radio-immunoprecipitation assay
ROI: Region of interest
RT-qPCR: Reverse transcription-quantitative polymerase chain reaction
SDS: Sodium dodecyl sulfate
SEM: Standard error of the mean
shRNA: Short hairpin RNA
STRING-db: STRING database
TA: Target activation
TEAB: Triethylammonium bicarbonate
TFA: Trifluoroacetic acid
TRIS: Tris(hydroxymethyl)aminomethane
UPLC: Ultra-performance liquid chromatography
WB: Western blotting
WHP: Woodchuck hepatitis virus
WPRE: Woodchuck hepatitis virus post-transcriptional regulatory element
