# Supplementary material for: Phosphoproteomic profiling reveals post-translational dysregulation in Huntington’s disease patient-derived neurons
Source: Cell Mol Biol Lett. 2026 Jun 2;31:129. doi: 10.1186/s11658-026-00948-2 (PMC13445741; doi:10.1186/s11658-026-00948-2)

1st Western blot of MXRA8 and P-MXRA8 labelled by native MXRA8 antibody

Uncropped blot with ladder

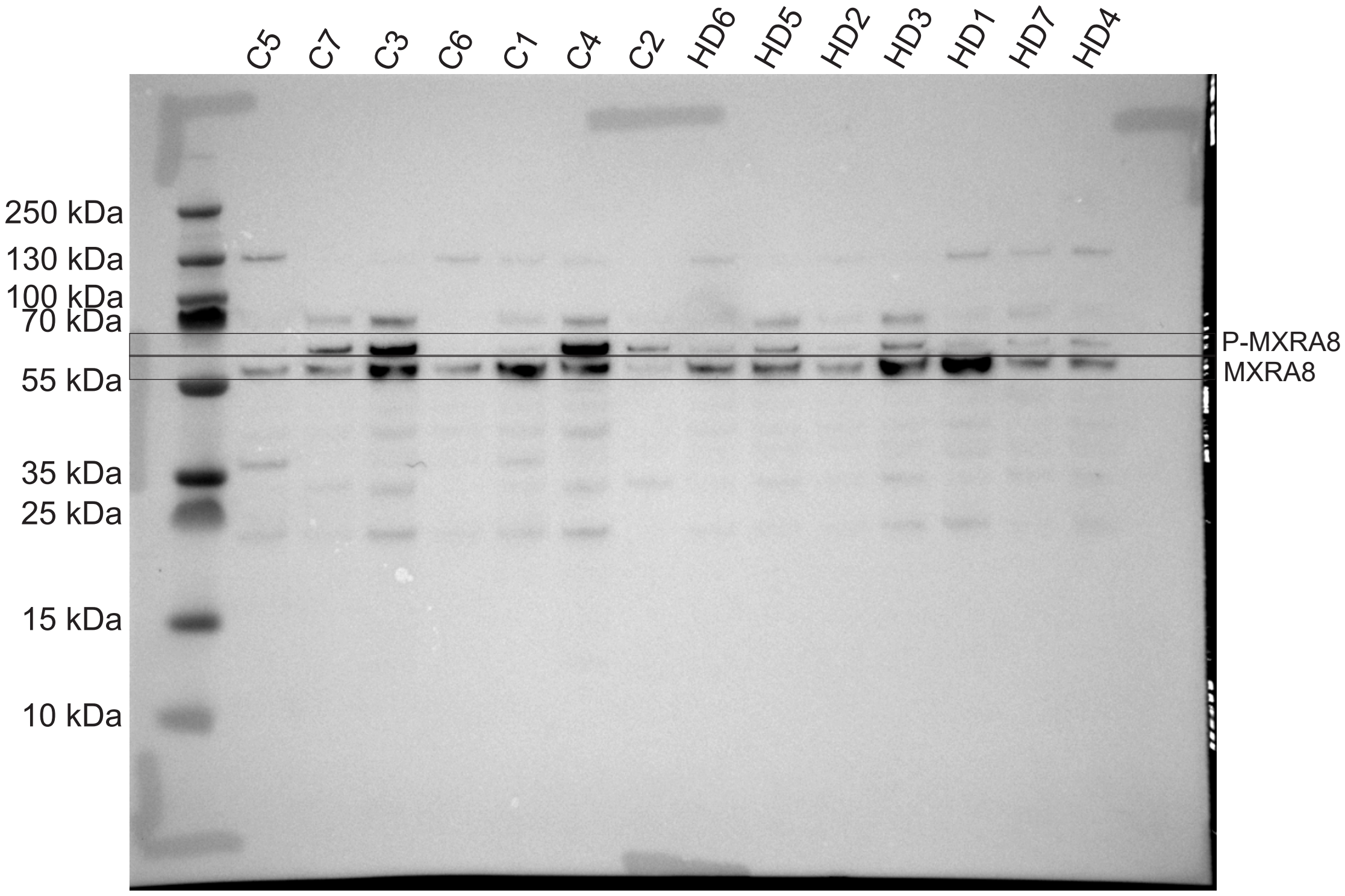

Uncropped blot without ladder

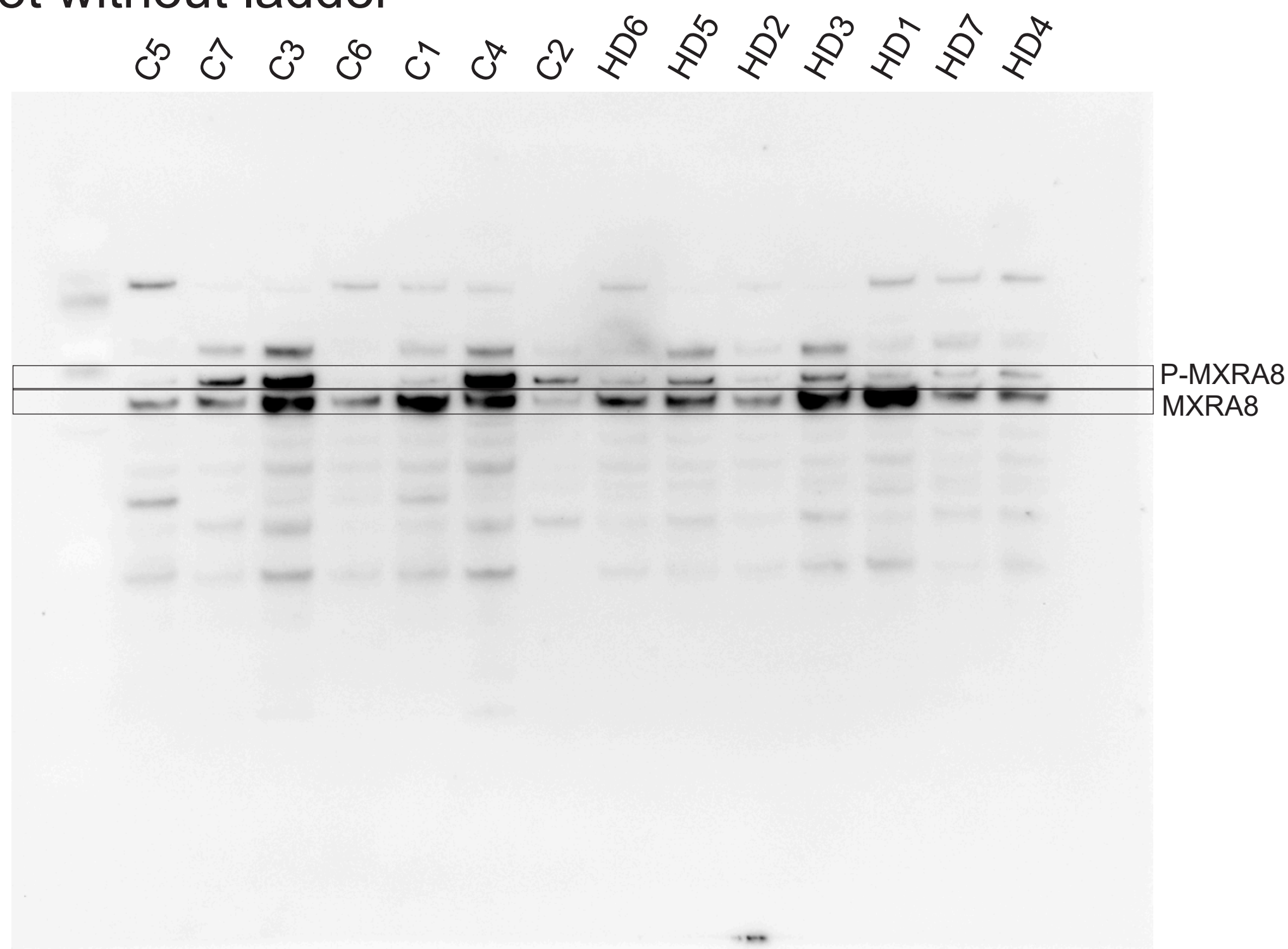

Actin without ladder

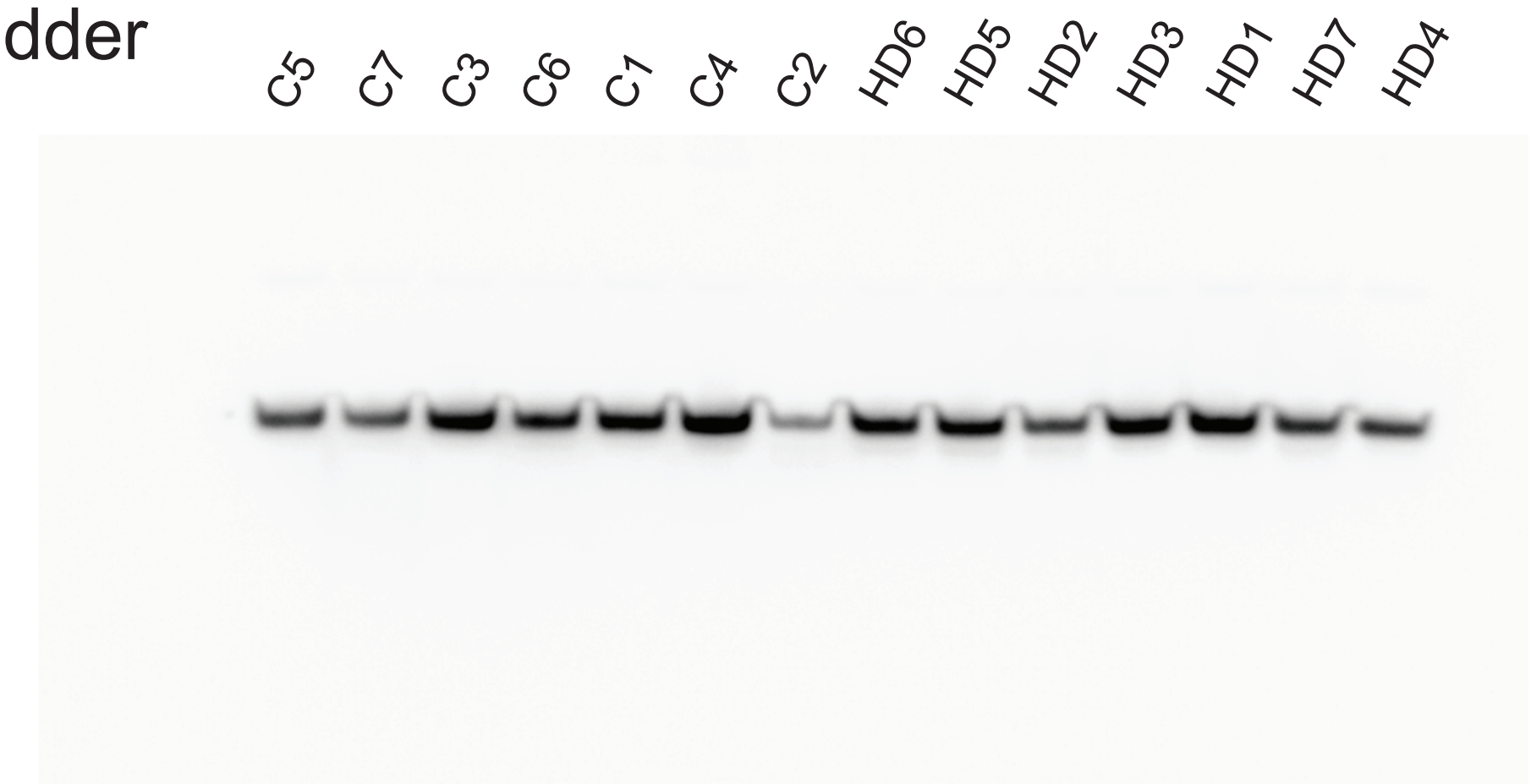

2nd Western blot of MXRA8 and P-MXRA8 labelled by native MXRA8 antibody

Uncropped blot with ladder

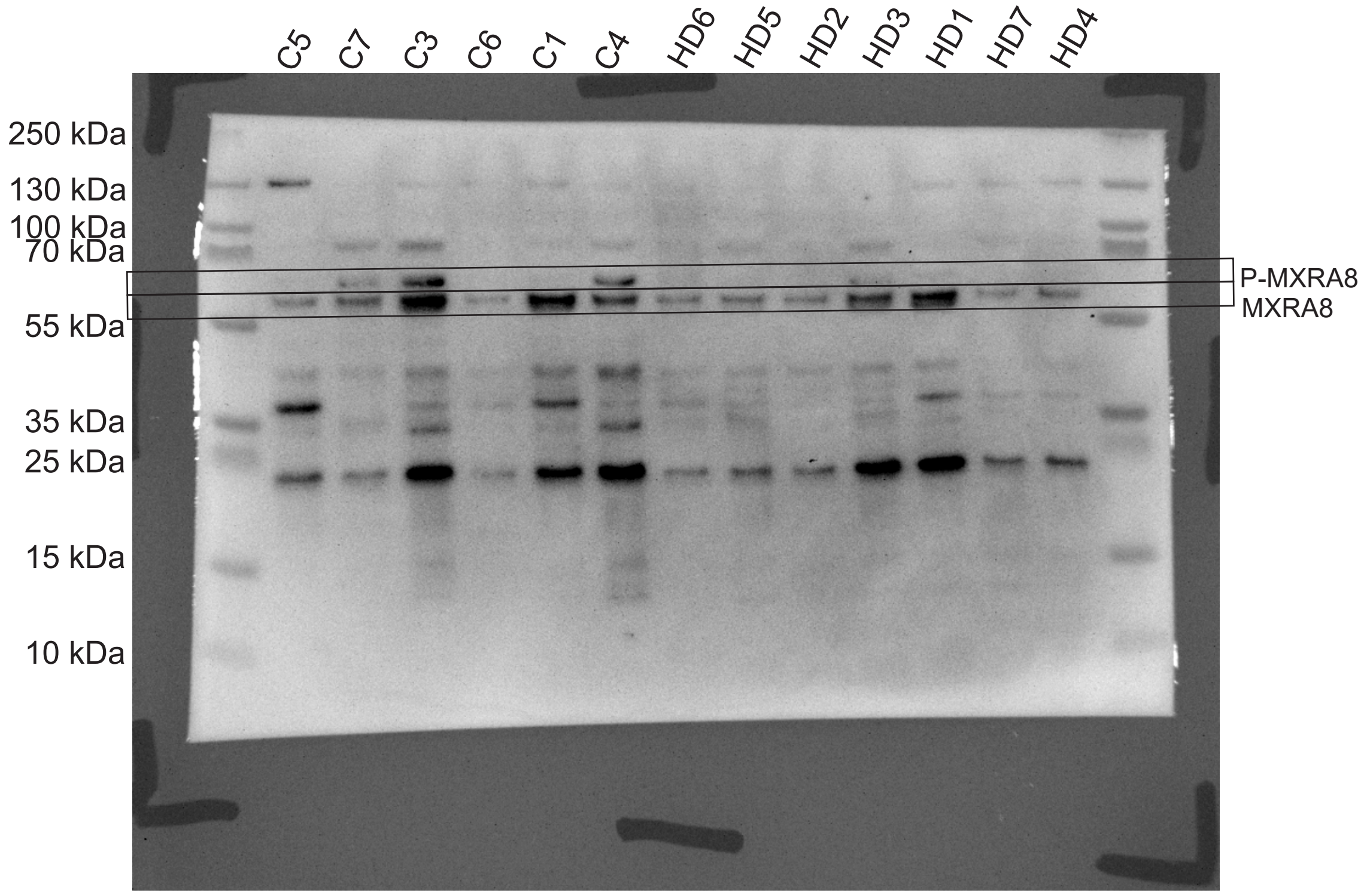

Uncropped blot without ladder

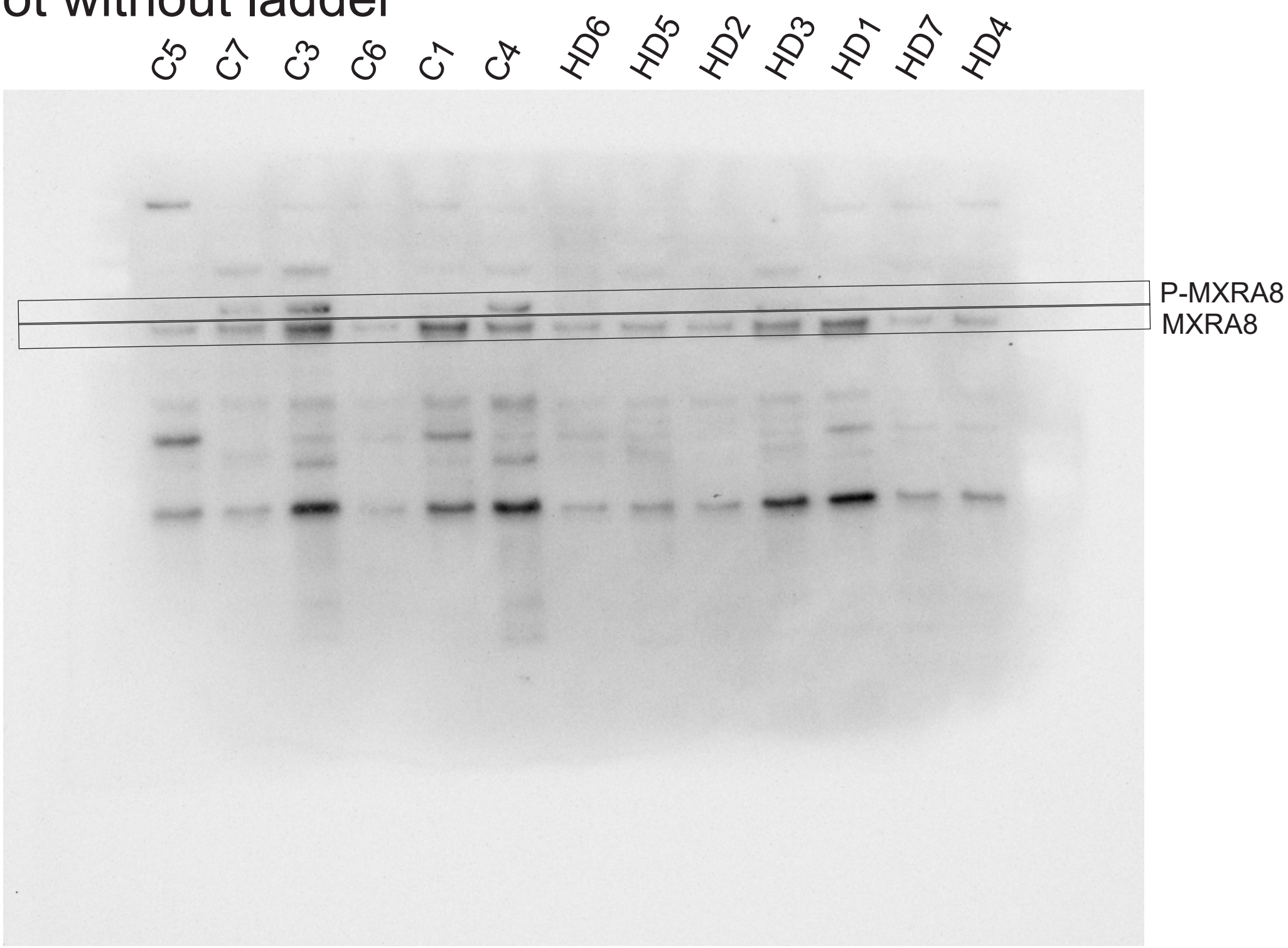

Actin without ladder

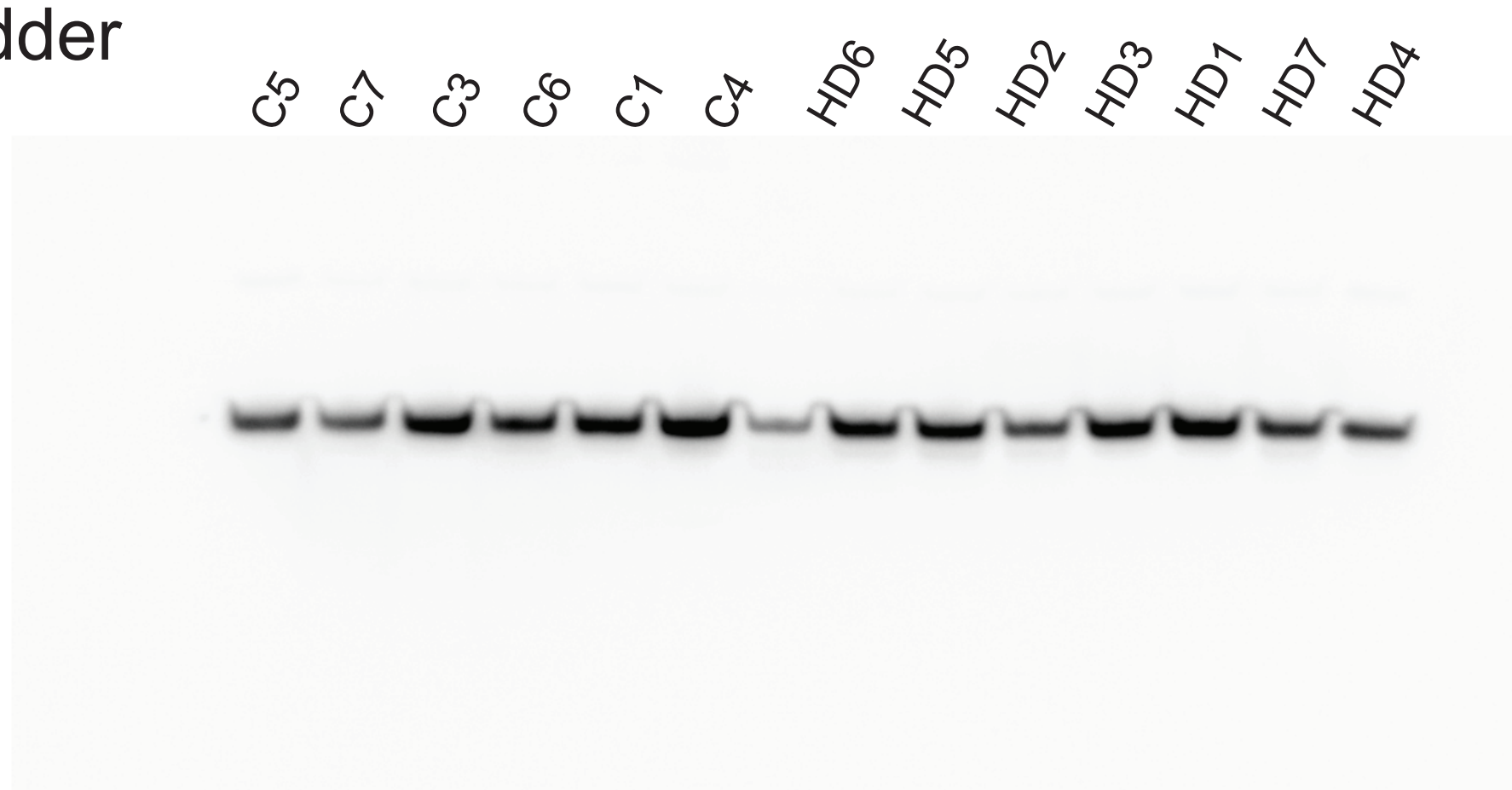

1st Western blot of P-MXRA8 labelled by S377 phospho-specific antibody

Uncropped blot with ladder

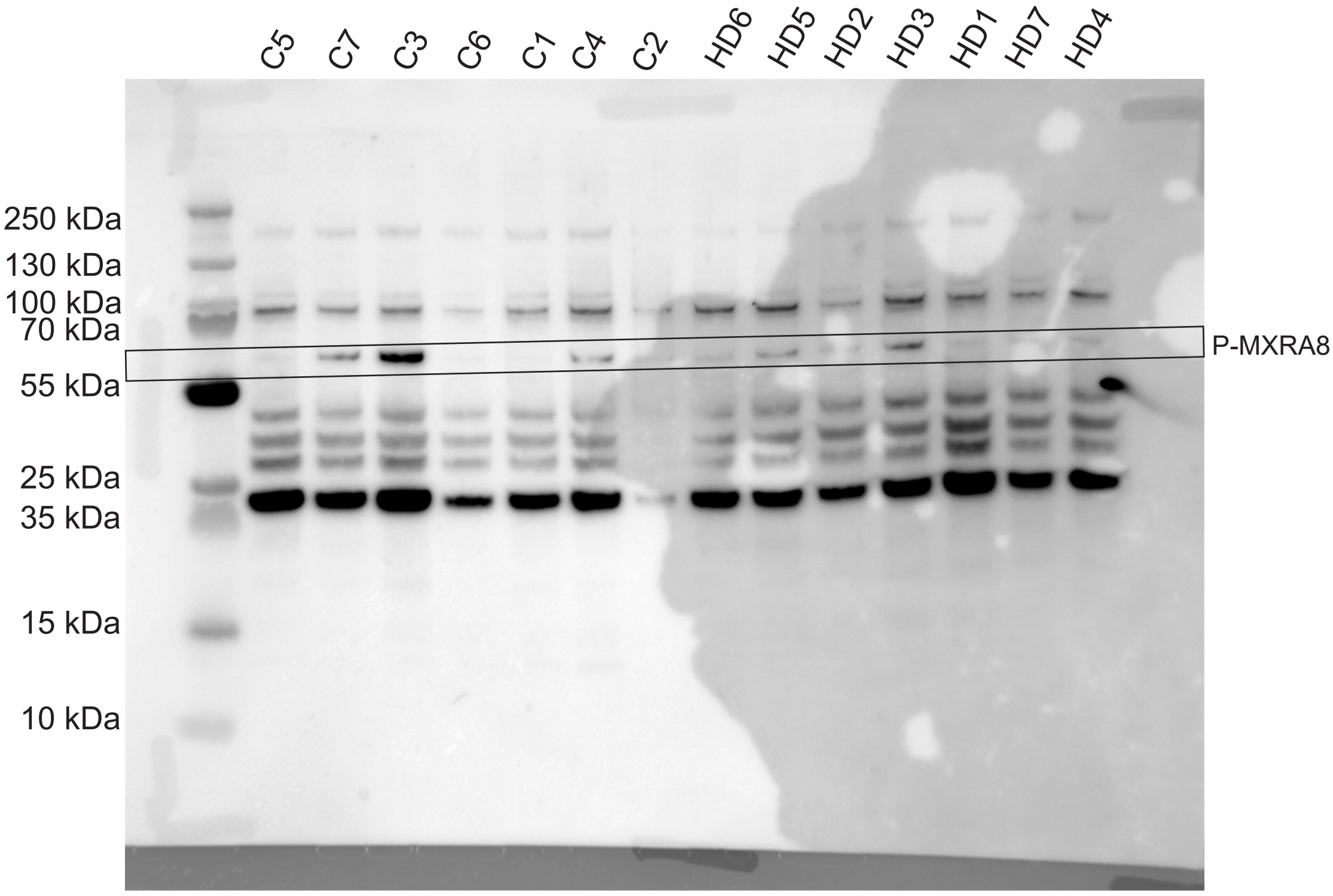

Uncropped blot without ladder

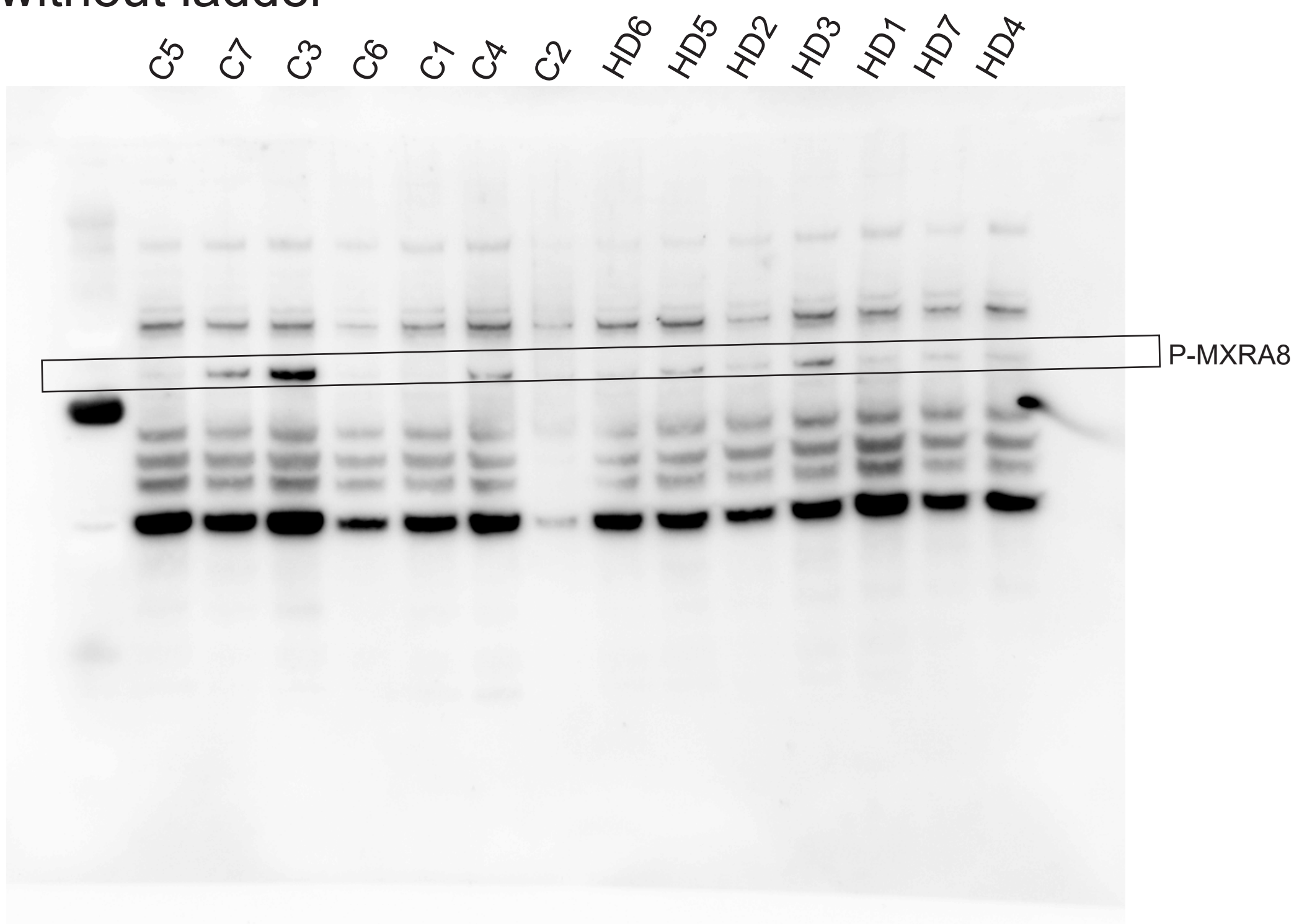

Actin without ladder

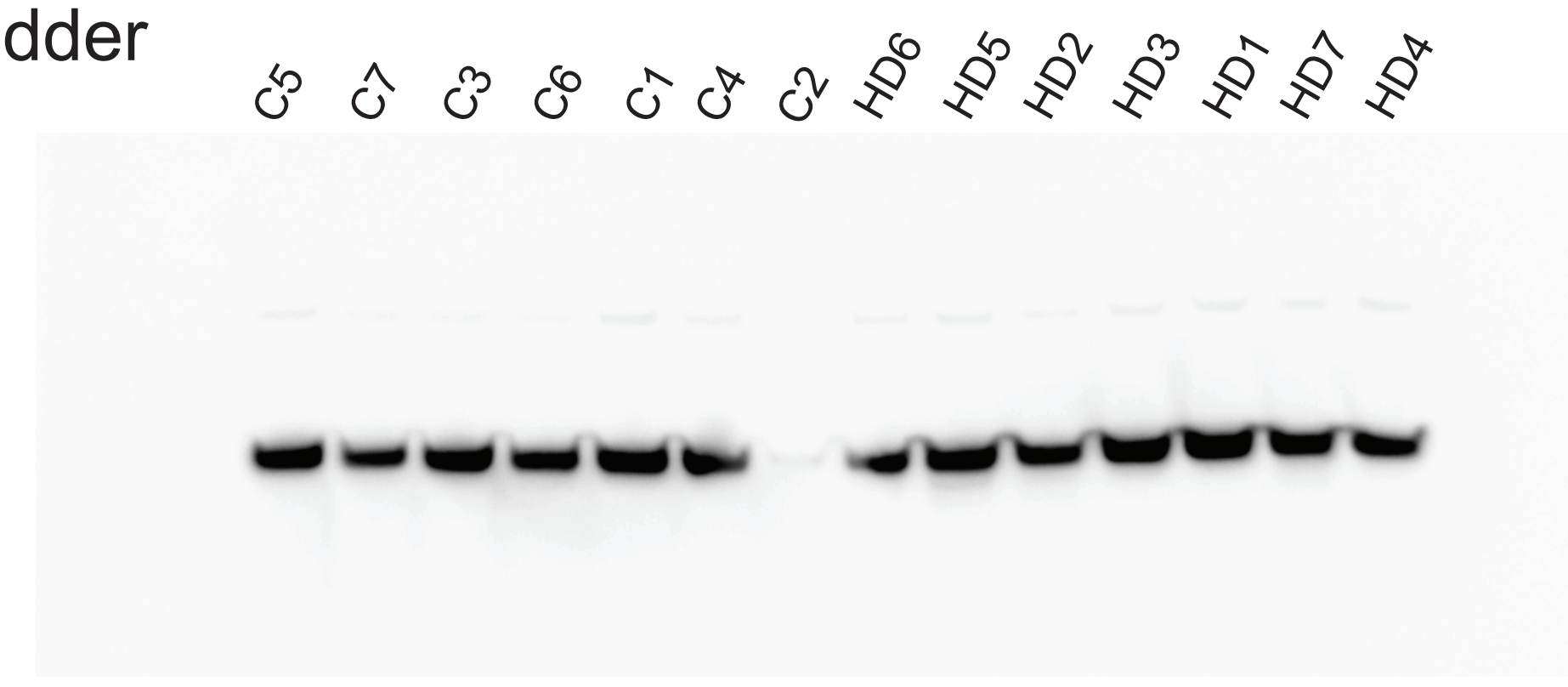

2nd Western blot of P-MXRA8 labelled by S377 phospho-specific antibody

Uncropped blot with ladder

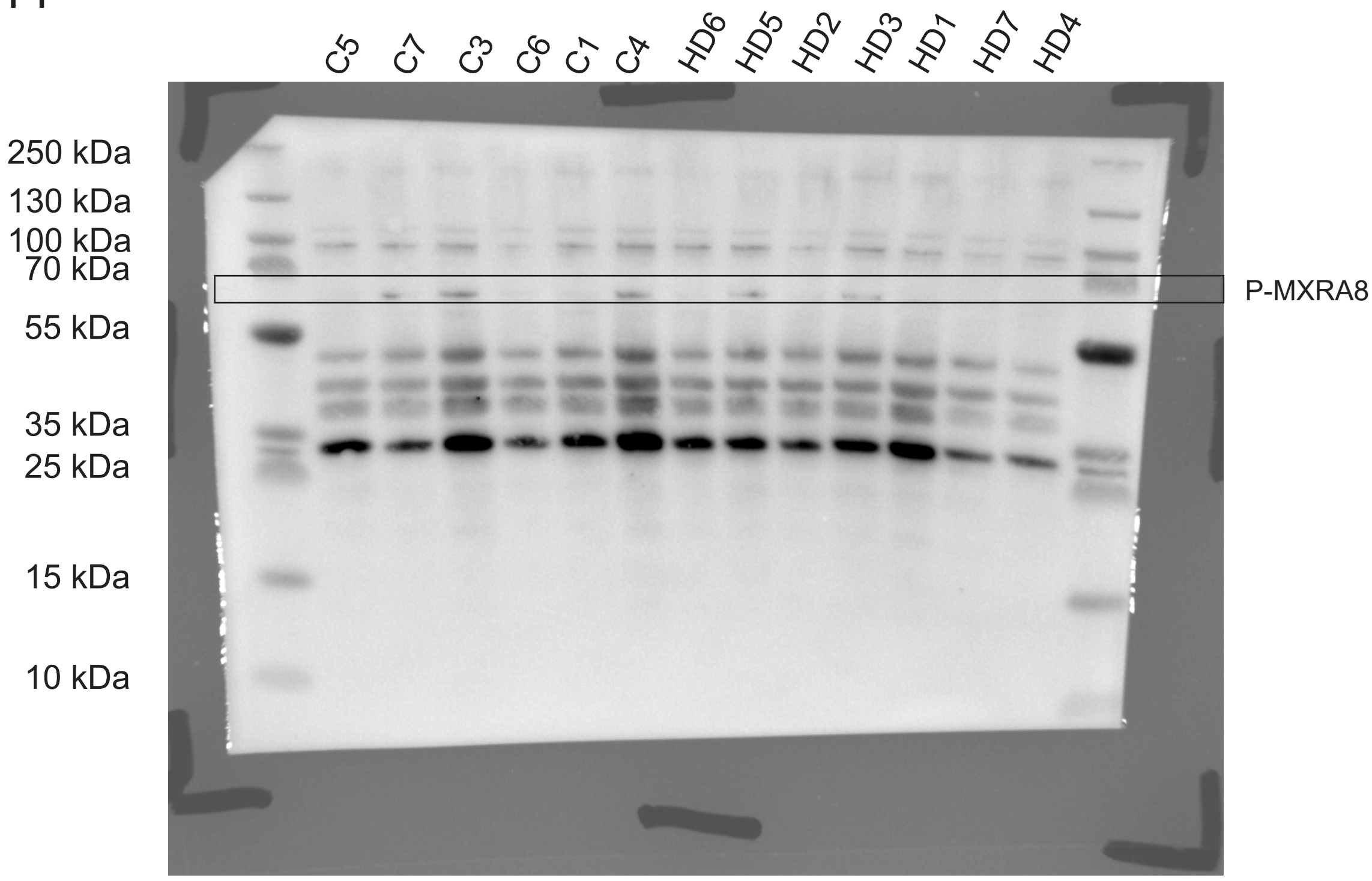

Uncropped blot without ladder

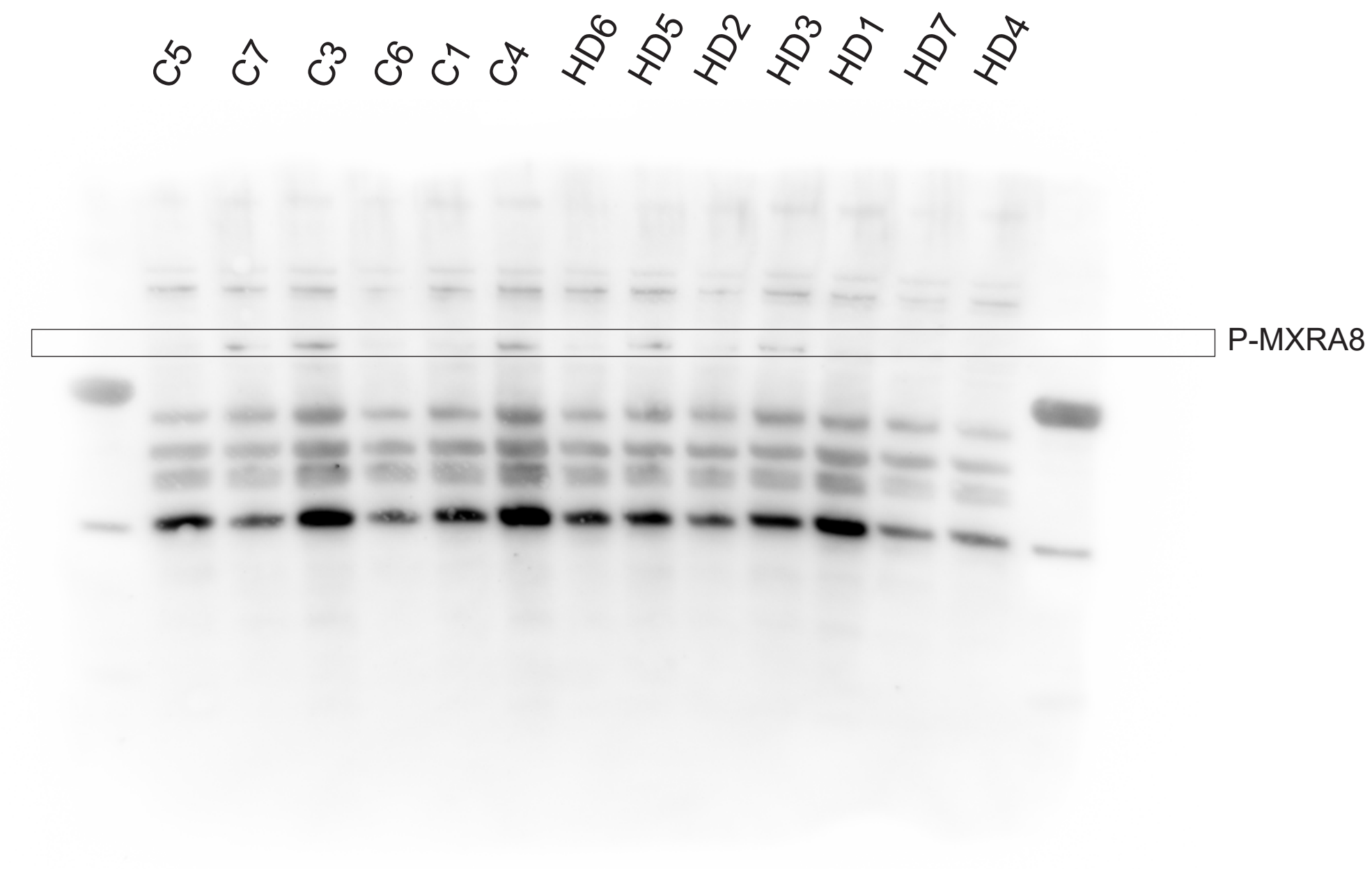

Actin without ladder

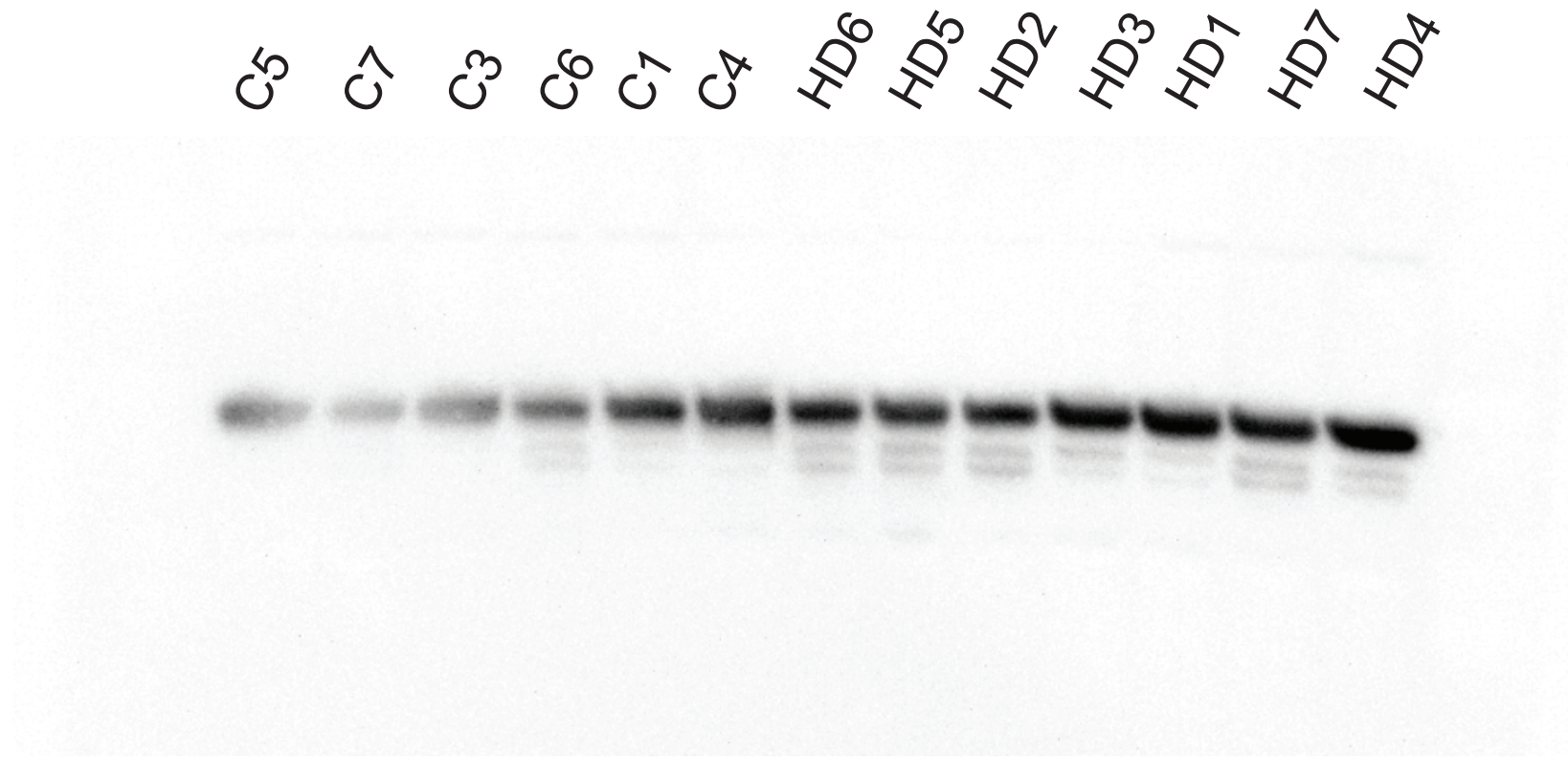

Western blot of MAPK9

Uncropped blot with ladder

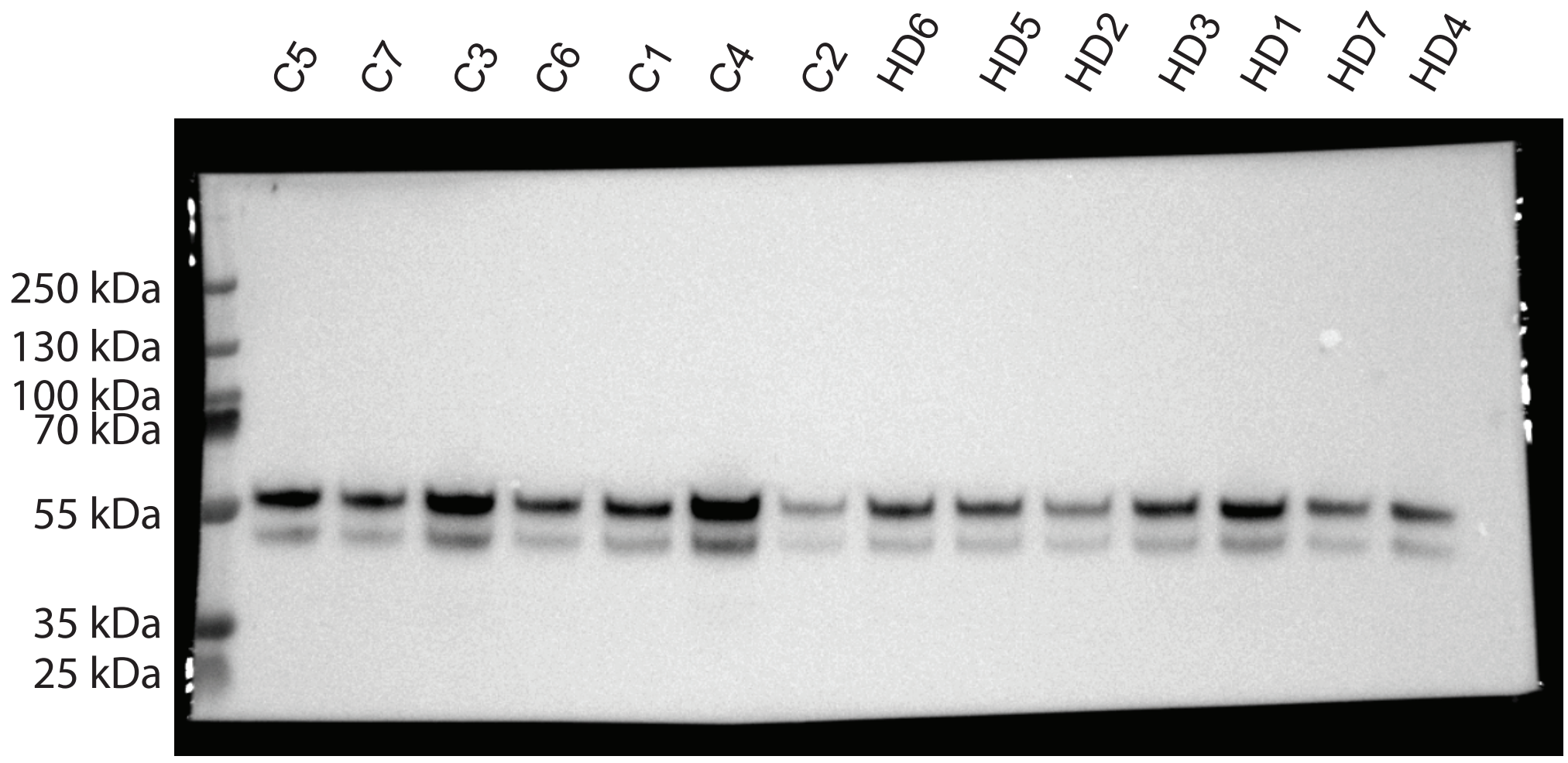

Uncropped blot without ladder

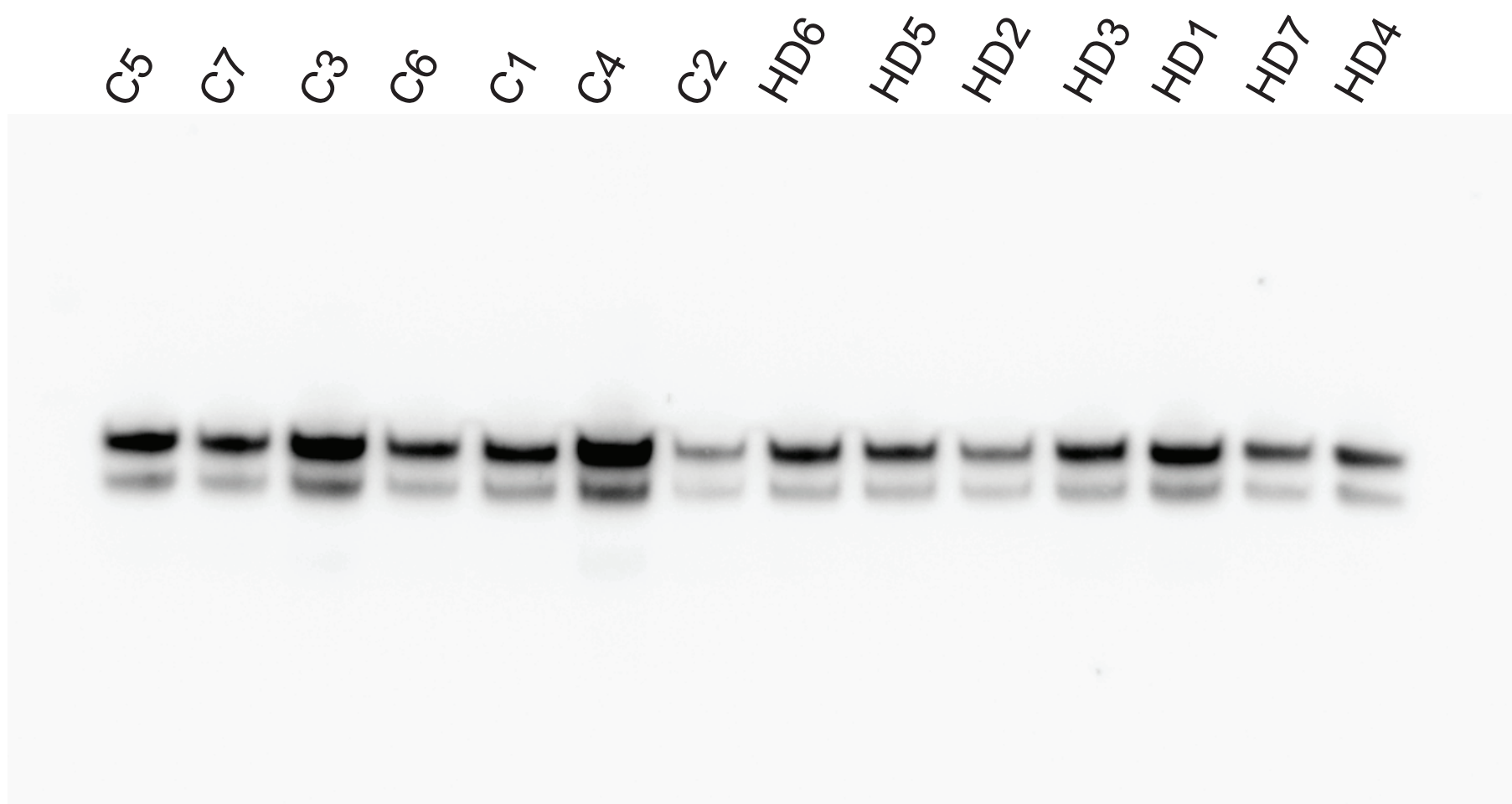

Actin without ladder

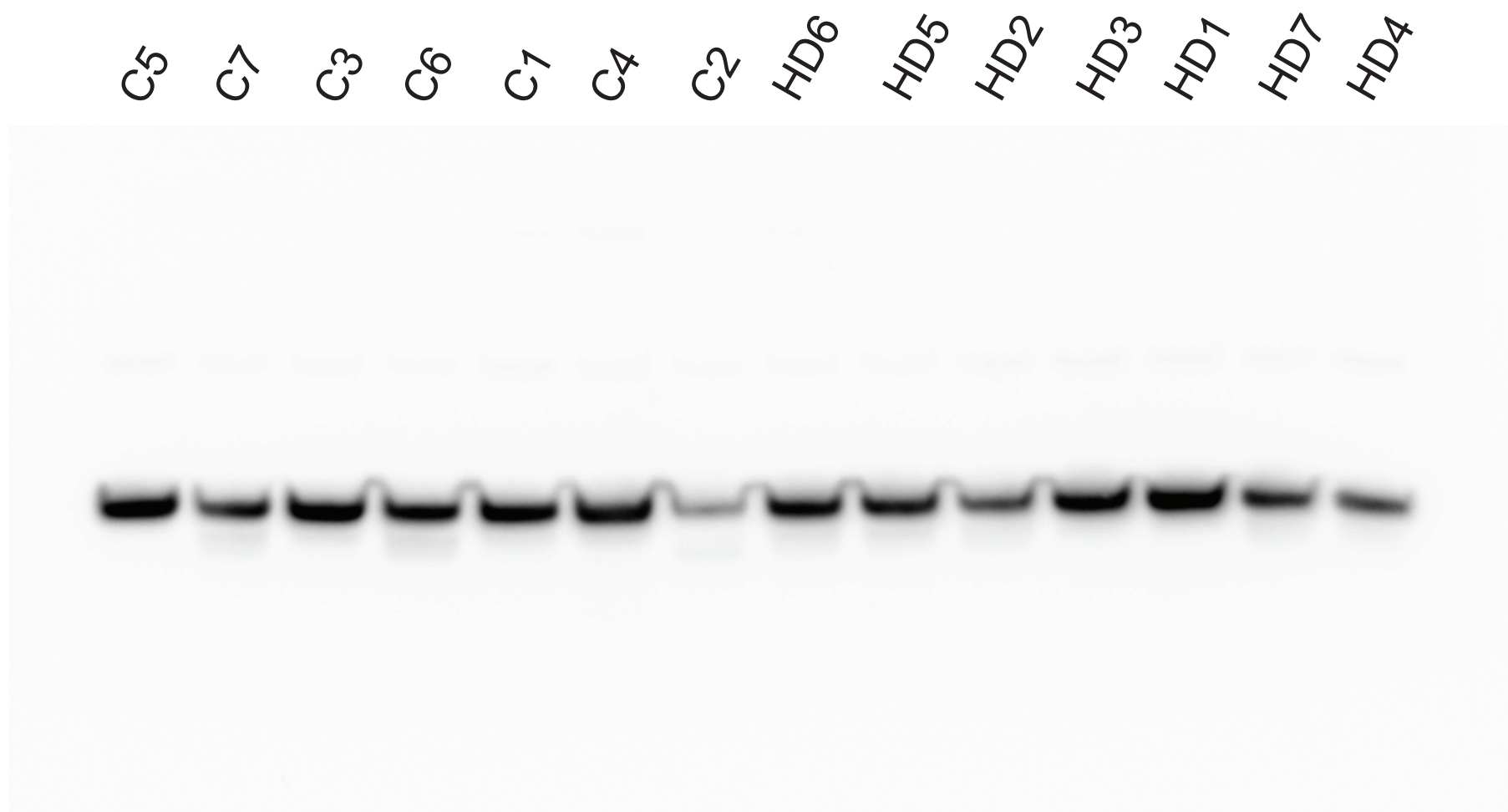

Western blot of STK10

Uncropped blot with ladder

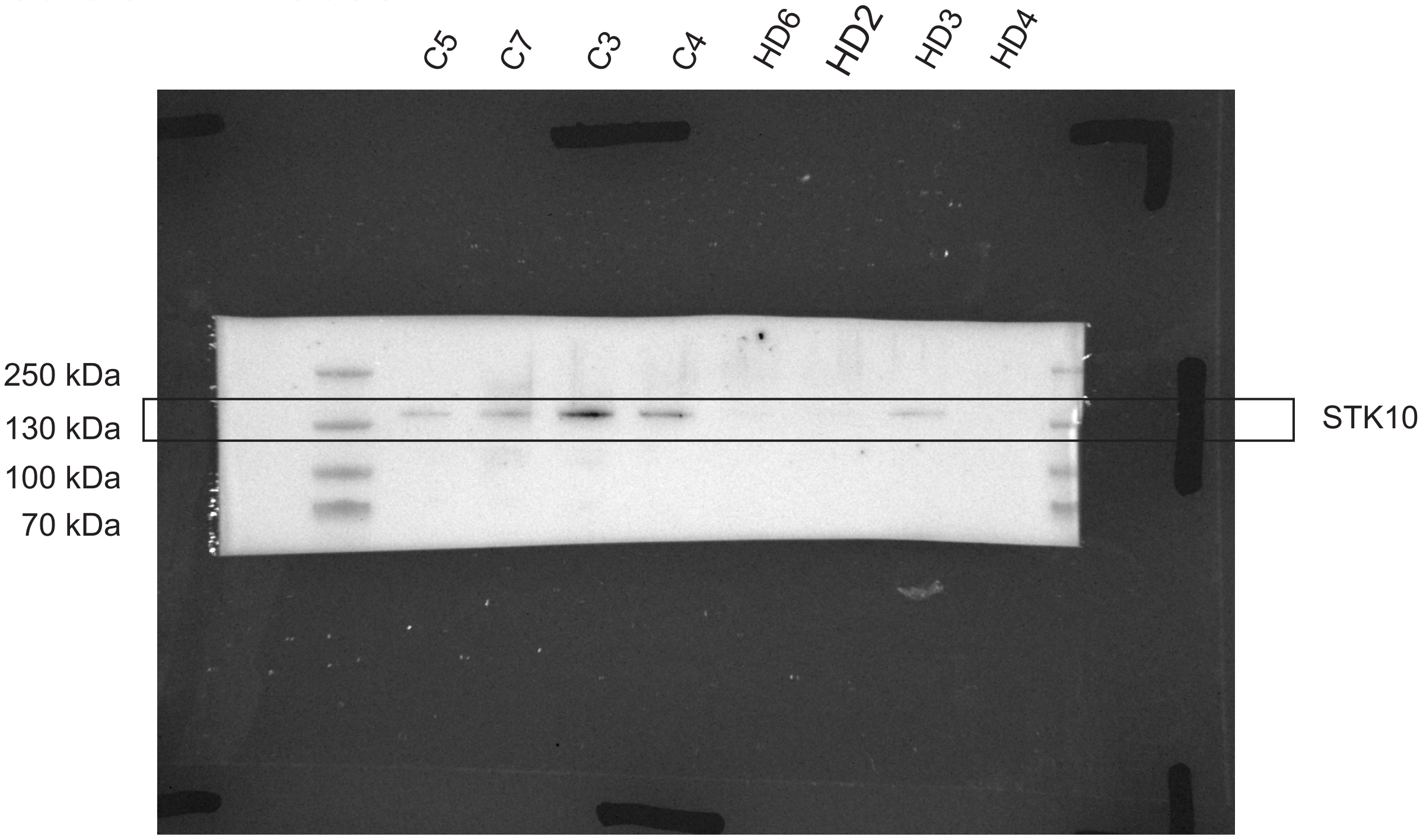

Uncropped blot without ladder

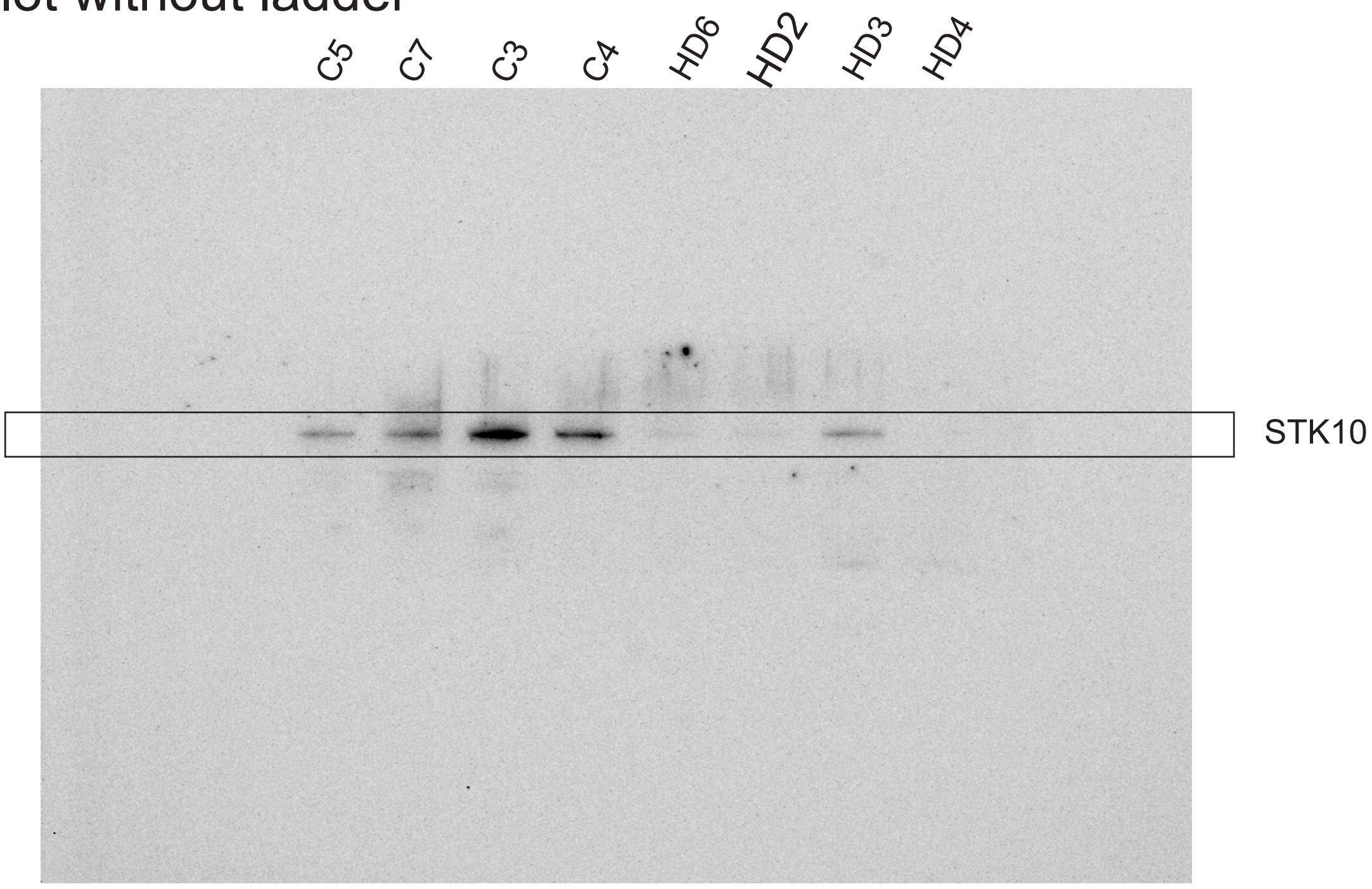

Actin without ladder

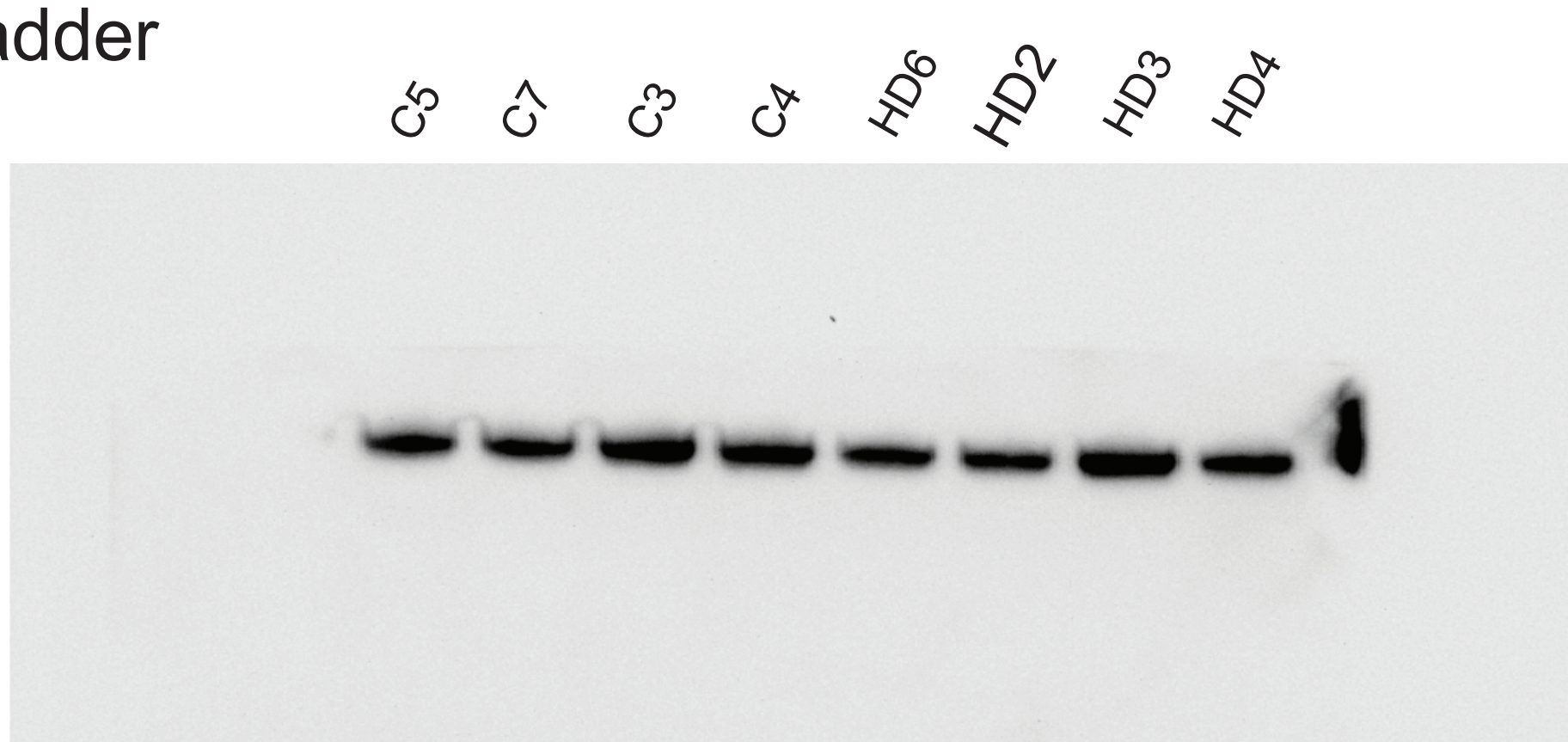

Western blot of EEF2K

Uncropped blot with ladder

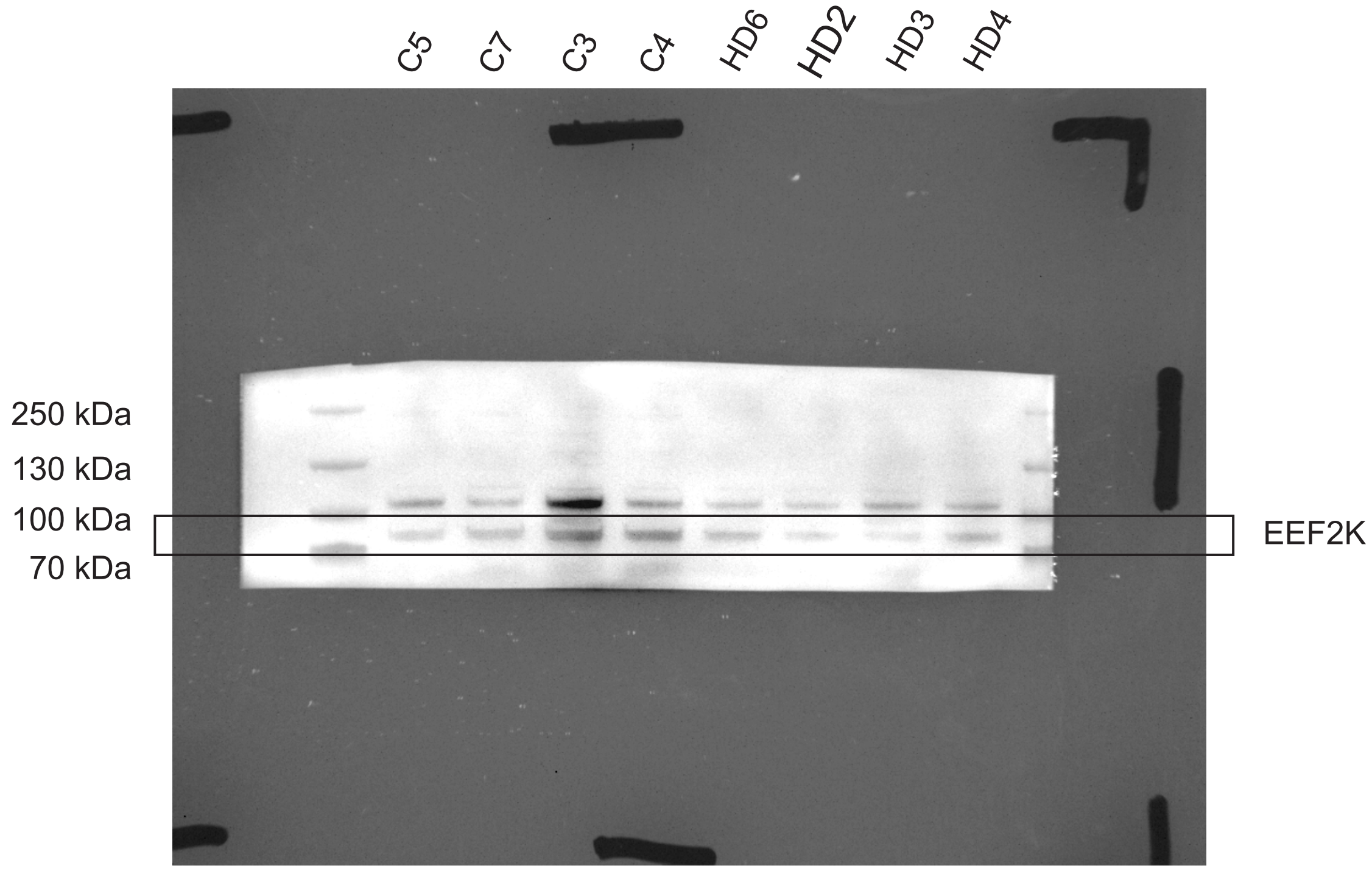

Uncropped blot without ladder

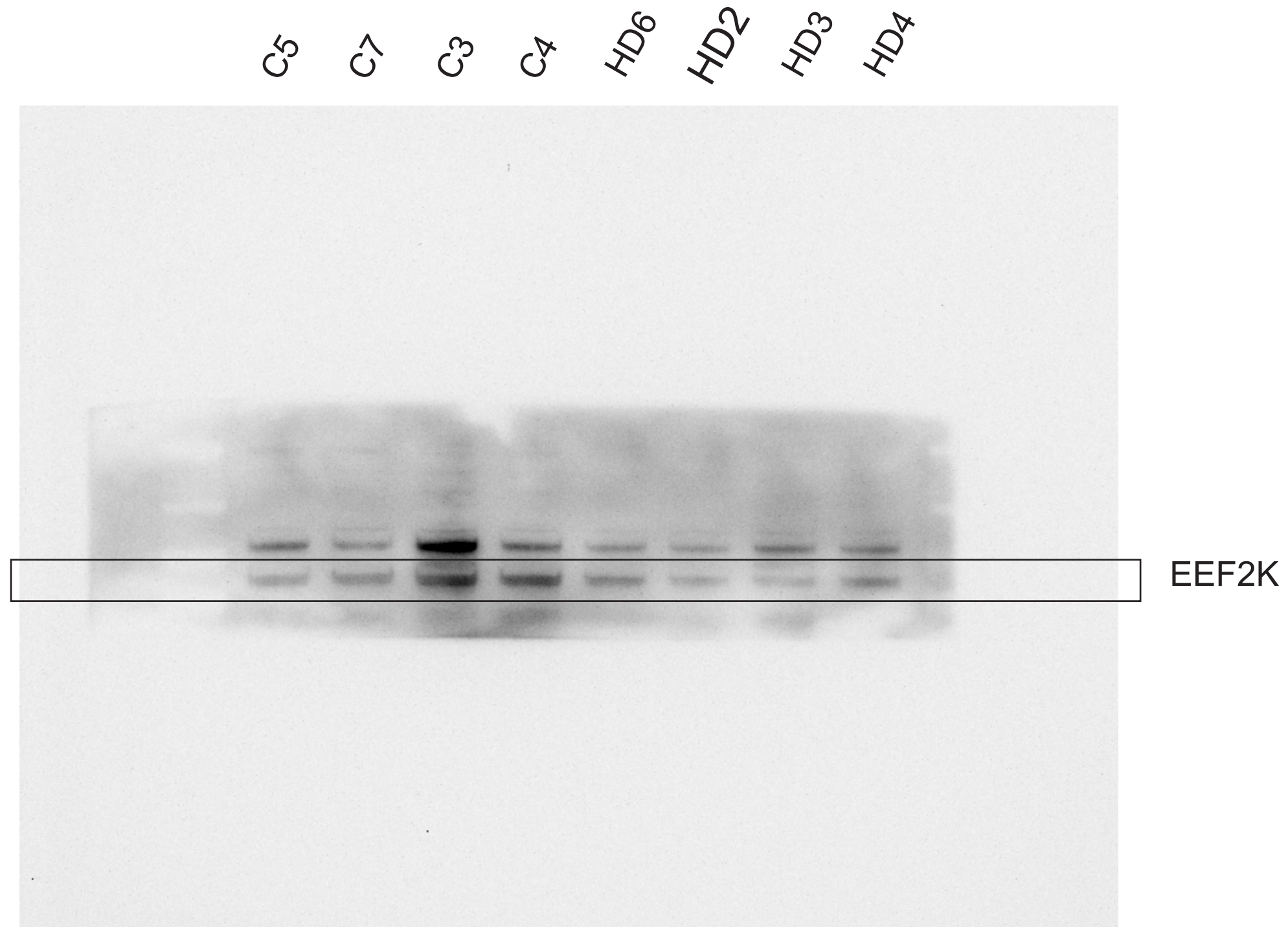

Actin without ladder

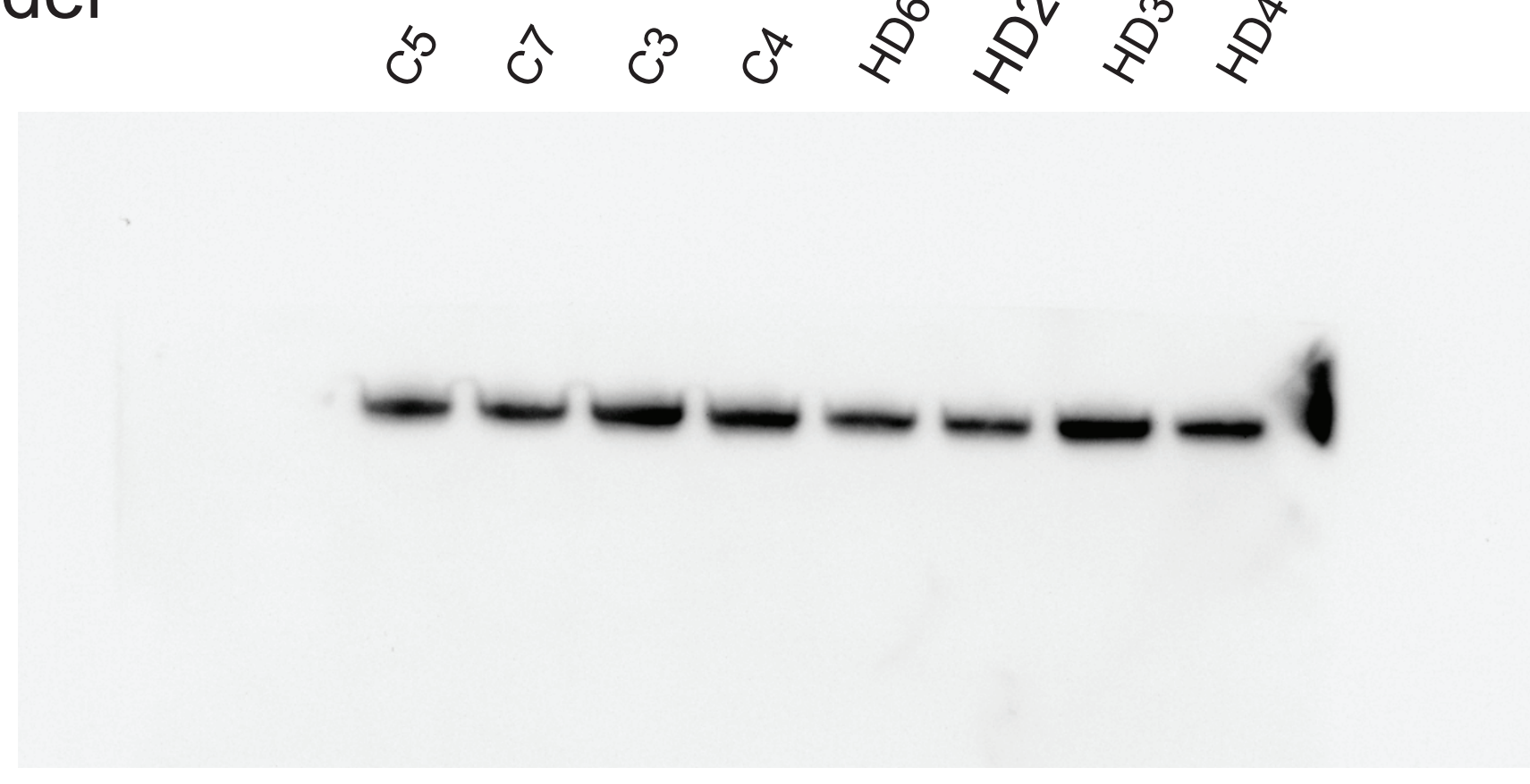

Supplement: Supplementary file 1 [file 11658_2026_948_MOESM1_ESM.pdf]
