# Supplementary material for: Phosphoproteomic profiling reveals post-translational dysregulation in Huntington’s disease patient-derived neurons
Source: Cell Mol Biol Lett. 2026 Jun 2;31:129. doi: 10.1186/s11658-026-00948-2 (PMC13445741; doi:10.1186/s11658-026-00948-2)
Supplement: Supplementary file 2 [file 11658_2026_948_MOESM2_ESM.pdf]

Supplementary Materials

List of contents

Supplementary Figure 1

Supplementary Figure 2

Supplementary Figure 3

Supplementary Figure 4

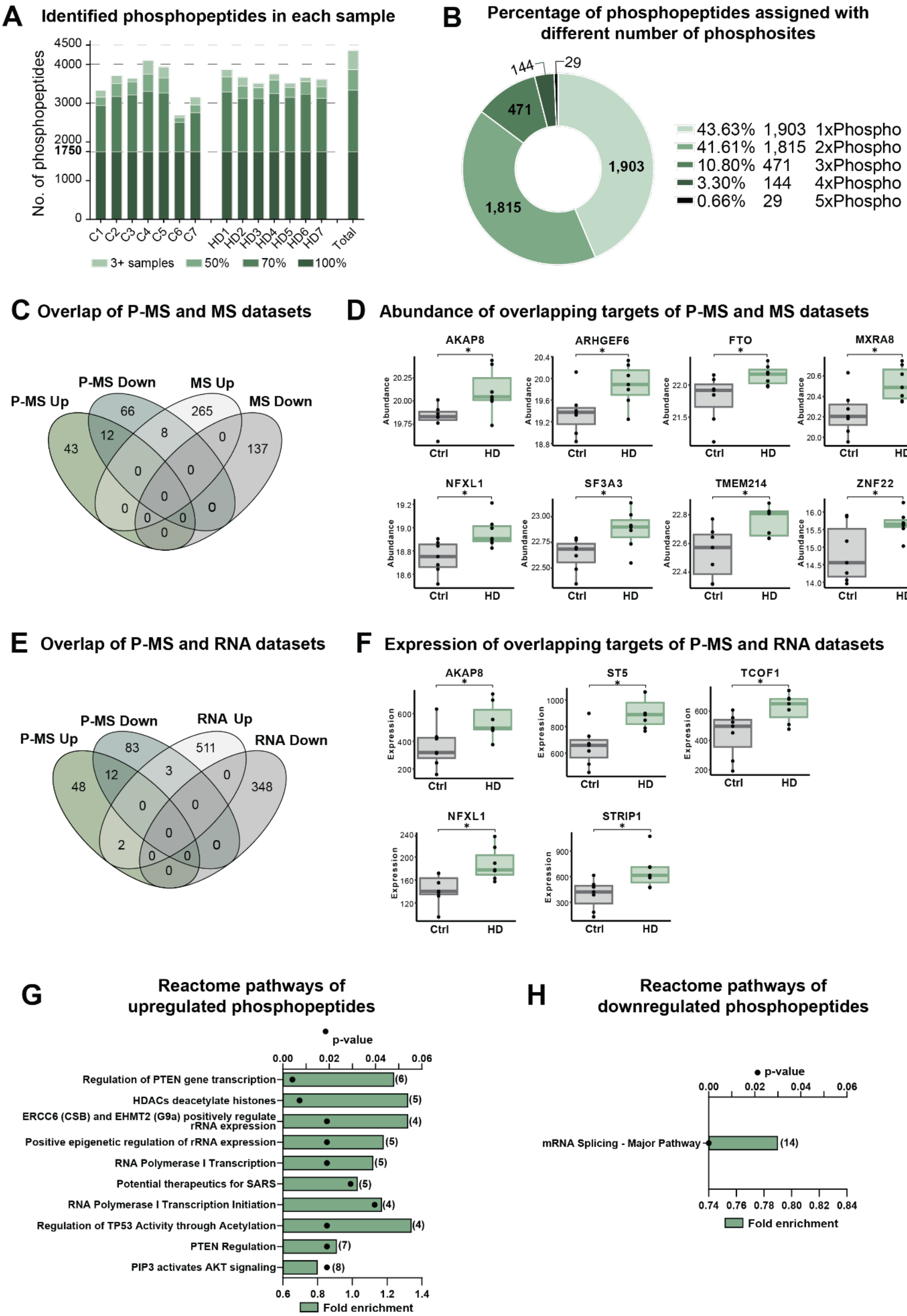

Supplementary Figure 1. Phosphoproteome of HD-iNs

(A) Bar plots showing the number of identified phosphopeptides separately in each sample as well as in total. 1,750 phosphopeptides were present in all the samples, while 1,589, 528 and 495 phosphopeptides could be found in at least 70%, 50% and 5% of the samples. (B) Diagram presents the percentage of phosphopeptides with 1, 2, 3, 4 or 5 assigned phosphosites of all phosphopeptides. (C) Venn diagram shows the number and percentage of overlapping targets of P-MS and MS datasets in iNs. (D) Box plots of the abundance of overlapping targets of P-MS and MS datasets in n=7 Ctrl- and n=7 HD-iN samples. (E) Venn diagram shows the number and percentage of the overlapping targets of P-MS and RNA-seq datasets. (F) Box plots of RNA expression of overlapping targets of P-MS and RNA datasets in n=7 Ctrl- and n=7 HD-iN samples. (G and H) Reactome pathways using STRING-db v12.0 of upregulated phosphopeptides (G) and downregulated phosphopeptides (H). Top ten most significant terms are shown. Bar plots represent fold enrichment (strength), dots represent Benjamini-Hochberg false discovery rates (n = 7 Ctrl- and 7 HD-iN samples, FDR<0.05).

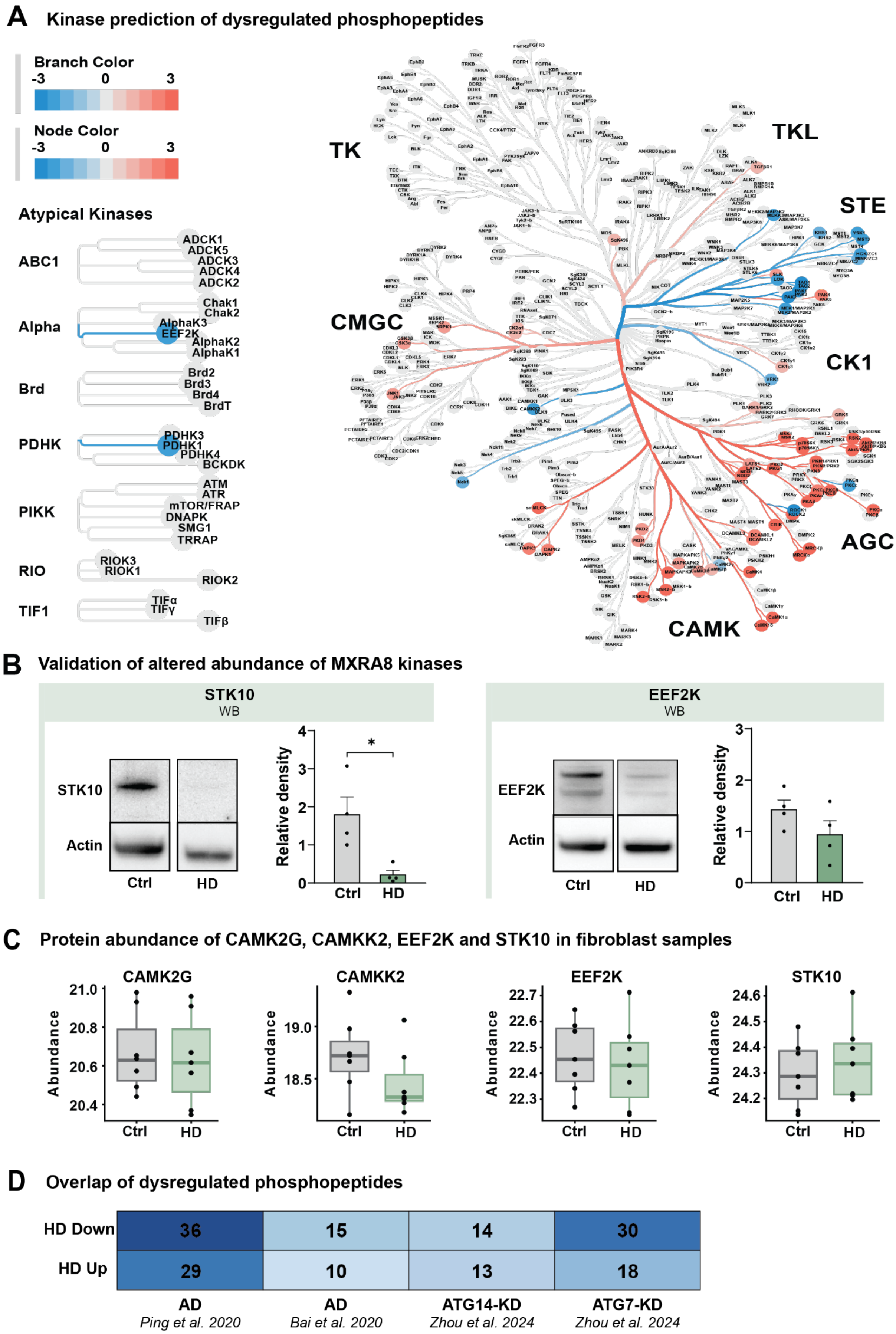
A Kinase prediction of dysregulated phosphopeptides
Branch Color
-3 0 3
Node Color
-3 0 3
Atypical Kinases
ABC1
ADCK1
ADCK5
ADCK3
ADCK4
ADCK2
Alpha
Chak1
Chak2
AlphaK3
EEF2K
AlphaK2
AlphaK1
Brd
Brd2
Brd3
Brd4
BrdT
PDHK
PDHK3
PDHK1
PDHK4
BCKDK
PIKK
ATM
ATR
mTOR/FRAP
DNAPK
SMG1
TRRAP
RIO
RIOK3
RIOK1
RIOK2
TIF1
TIFα
TIFγ
TIFβ
TK
TKL
STE
CMGC
CK1
AGC
CAMK
B Validation of altered abundance of MXRA8 kinases
STK10
WB
STK10
Actin
Ctrl
HD
Relative density
0 1 2 3 4
*
Ctrl
HD
EEF2K
WB
EEF2K
Actin
Ctrl
HD
Relative density
0 1 2 3
Ctrl
HD
C Protein abundance of CAMK2G, CAMKK2, EEF2K and STK10 in fibroblast samples
CAMK2G
Abundance
20.4 20.6 20.8 21.0
Ctrl
HD
CAMKK2
Abundance
18.5 19.0
Ctrl
HD
EEF2K
Abundance
22.3 22.4 22.5 22.6 22.7
Ctrl
HD
STK10
Abundance
24.2 24.3 24.4 24.5 24.6
Ctrl
HD
D Overlap of dysregulated phosphopeptides
HD Down 36 15 14 30
HD Up 29 10 13 18
AD
Ping et al. 2020
AD
Bai et al. 2020
ATG14-KD
Zhou et al. 2024
ATG7-KD
Zhou et al. 2024

Supplementary Figure 2. Identification of kinases of dysregulated phosphopeptides in HD-iNs and overlap of dysregulated phosphopeptides.

(A) Kinase tree showing the kinases predicted to be differentially active in the HD-iNs when compared to the Ctrl-iNs. (B) Abundance of STK10 (left) and EEF2K (right) by western blot experiments in n=4 Ctrl- and n=4 HD-iN samples. Blot sections show one representative Ctrl- and HD-iN sample. Bar plots show the relative density of the proteins. (C) Box plots showing the abundance of CAMK2G, CAMKK2, EEF2K and STK10 proteins measured by MS in parental fibroblasts corresponding to the iN lines. (D) Heatmap of overlaps with AD and autophagy-related phosphoproteins. (B, C) In panel B, samples are from converted iN cultures; in panel C, samples are from the corresponding parental fibroblasts. Data is shown as mean ± SEM in B. Center lines indicate the median, boxes indicate the interquartile range, and whiskers indicate the range in C. Data is shown as mean ± SEM in B. Statistical analysis was performed using two-tailed t-tests in all cases; * $p<0.05$.

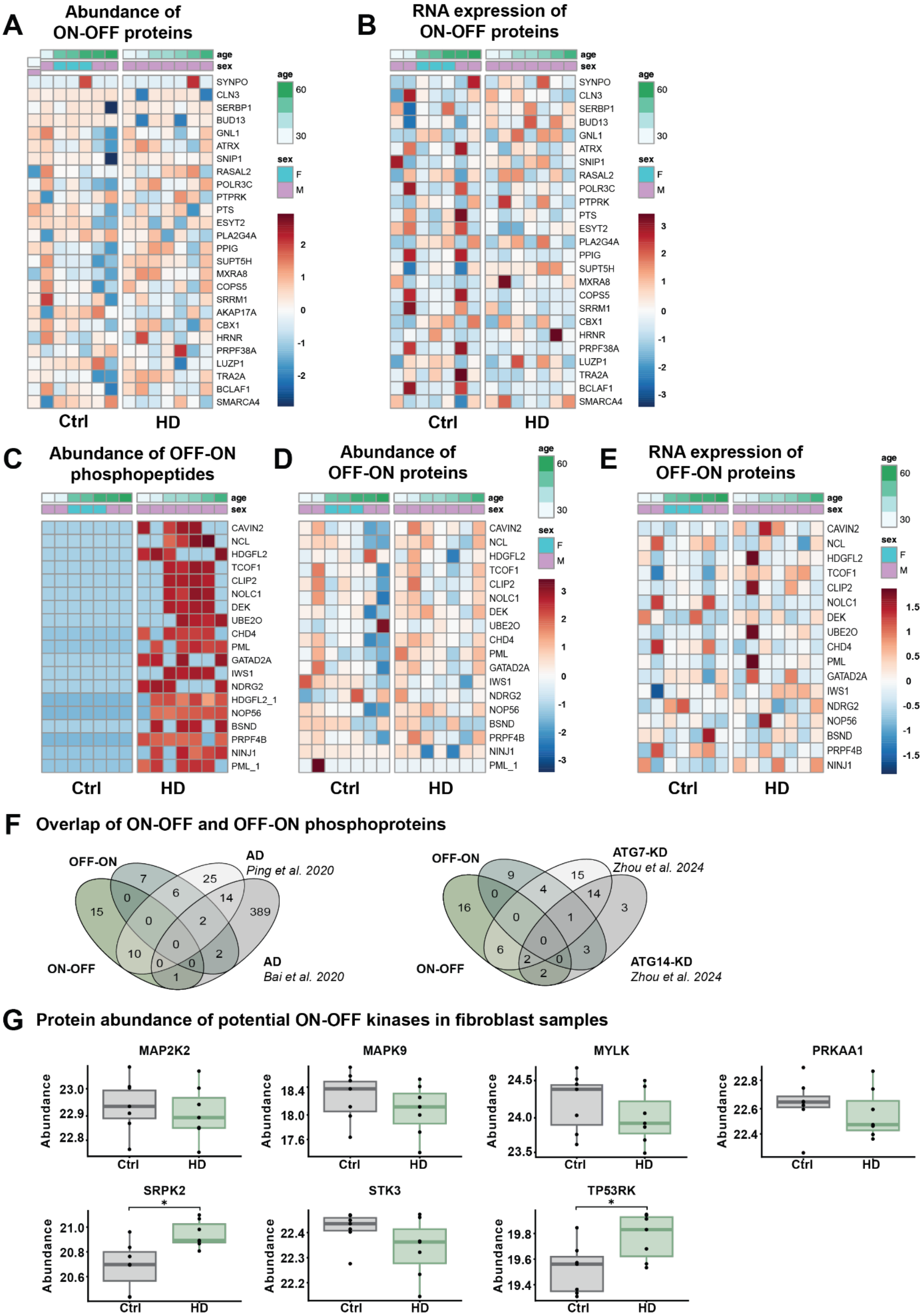

Supplementary Figure 3. ON-OFF and OFF-ON phosphoproteins in HD-iNs.

A) Heatmap of ON-OFF protein abundance in the proteomic dataset of Ctrl- and HD-iNs. (B) Heatmap of gene expression of the ON-OFF proteins in the bulk-RNAseq data of Ctrl- and HD-iNs. (C) Heatmap showing the abundance of the OFF-ON phosphopeptides in each sample. (D) Heatmap of OFF-ON protein abundance in the proteomic data of Ctrl- and HD-iNs. (E) Heatmap of gene expression of the OFF-ON proteins in the bulk-RNAseq data of Ctrl- and HD-iNs. (F) Venn diagrams showing the number of intersections of proteins associated with ON-OFF and OFF-ON phosphopeptides in the HD-iNs with those seen in (left) autophagy disruption experiments in hiPSC-iNs, and (right) studies of Alzheimer's disease in post mortem frontal cortex samples. (G) Box plots showing the abundance of the identified putative ON-OFF kinases measured by MS in parental fibroblasts. Each dot represents one Ctrl or one HD adult human fibroblast cell line. Center lines indicate the median, boxes indicate the interquartile range, and whiskers indicate the range. Statistical analysis was performed using two-tailed t-tests in all cases; * $p<0.05$.

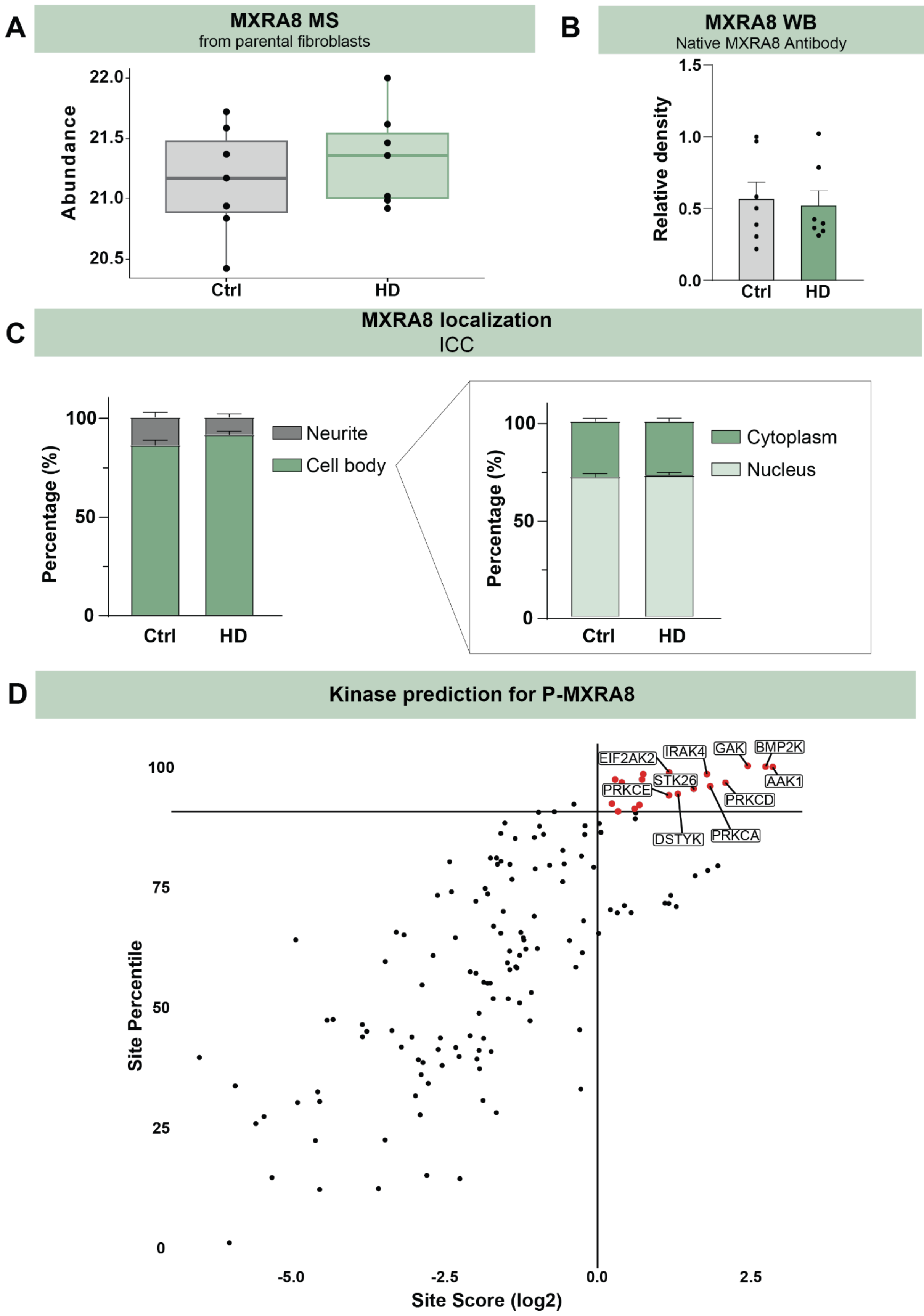
A
MXRA8 MS
from parental fibroblasts
Abundance
22.0
21.5
21.0
20.5
Ctrl
HD
B
MXRA8 WB
Native MXRA8 Antibody
Relative density
1.5
1.0
0.5
0.0
Ctrl
HD
C
MXRA8 localization
ICC
Percentage (%)
100
50
0
Ctrl
HD
Neurite
Cell body
Cytoplasm
Nucleus
D
Kinase prediction for P-MXRA8
Site Percentile
Site Score (log2)
100
75
50
25
0
-5.0
-2.5
0.0
2.5
EIF2AK2
IRAK4
GAK
BMP2K
AAK1
STK26
PRKCE
PRKCD
PRKCA
DSTYK

Supplementary Figure 4. Abundance and localization of MXRA8 and its binding partners.

(A) Box plot showing MXRA8 protein abundance measured by MS in parental fibroblasts corresponding to the iN lines. (B) Abundance of the MXRA8 protein using native MXRA8 antibody in n=14, N=7 Ctrl- and n=14, N=7 HD-iNs. (C) Localization of MXRA8 by HCA in TAU counterstained N=7 Ctrl- and N=7 HD-iN samples. Bars on the left show the percentage of the MXRA8^{+} dots in the cell body versus in the neurites, and on the right, in the cytoplasm versus the nucleus, of Ctrl- and HD-iNs. (D) Scatter plot showing the kinase prediction results for MXRA8-pS377 from PhosphositePlus, with the Site Score on the X-axis and Site Percentile on the Y-axis. The points marked in red show the significant results, while the remaining represent the non-significant results. Any kinase with a site percentile > 90 and site score (log2) > 0, and identified in the proteomic dataset, was considered significant. (A, B) Each dot represents one Ctrl or one HD adult human cell line. In panel A, samples are from the corresponding parental fibroblasts; in panel B, samples are from converted iN cultures. Center lines indicate the median, boxes indicate the interquartile range, and whiskers indicate the range in A. Data is shown as mean ± SEM in B and C.Statistical analysis was performed using two-tailed t-tests in all cases; * $p<0.05$.
